# Extra Virgin Olive Oil: Molecular Mechanisms, Bioavailability Challenges, and Therapeutic Perspectives

**DOI:** 10.3390/nu18152416

**Published:** 2026-07-24

**Authors:** Muhammad Maaz, Muhammad Tauseef Sultan, Ahmad Mujtaba Noman, Ralf Weiskirchen, Waleed Rizk ElGhareeb, Bodour Ibrahim Al Shik Mubarak, Adel A. Rezk, Marwa Ezz El-Din Ibrahim

**Affiliations:** 1 Department of Human Nutrition, Faculty of Food Science and Nutrition, Bahauddin Zakariya University, Multan 60800, Pakistan; maazm4158@gmail.com (M.M.); tauseefsultan@bzu.edu.pk (M.T.S.); ahmadmujtaba493@gmail.com (A.M.N.); 2 Institute of Molecular Pathobiochemistry, Experimental Gene Therapy and Clinical Chemistry (IFMPEGKC), RWTH University Hospital Aachen, 52074 Aachen, Germany; 3 Department of Public Health, College of Veterinary Medicine, King Faisal University, Al-Ahsa 31982, Saudi Arabia; welsaid@kfu.edu.sa; 4 Department of Food and Nutrition Science, College of Agricultural and Food Sciences, King Faisal University, Al-Ahsa 31982, Saudi Arabia; bimubarak@kfu.edu.sa; 5 Agricultural Biotechnology Department, College of Agricultural and Food Science, King Faisal University, Al-Ahsa 31982, Saudi Arabia; arazk@kfu.edu.sa

**Keywords:** extra virgin olive oil, olive phenolics, bioavailability, anticancer, apoptosis induction, signaling pathways, preclinical evidence

## Abstract

**Background/Objectives**: Extra virgin olive oil (EVOO), a key component of the Mediterranean diet, has attracted research interest because olive-derived phenolics demonstrate potential anticancer activity in experimental models. This review summarizes evidence concerning whole EVOO, phenolic-enriched EVOO, olive phenolic extracts, and the isolated compounds hydroxytyrosol, oleuropein, oleocanthal, and oleacein. **Methods**: A structured narrative search of PubMed, Web of Science, ScienceDirect, and Google Scholar was conducted for literature published between 2015 and 2025. Evidence was reviewed for breast, prostate, colorectal, pancreatic, bone, oral, liver, gastric, hematological, and brain cancers. Comparatively limited evidence concerning cervical, endometrial, ovarian, melanoma, non-melanoma skin, and thyroid cancers was summarized separately. **Results**: The molecular evidence was derived primarily from cell culture and animal studies using isolated phenolics and concentrated extracts. Preclinical studies indicate that EVOO phenolics may demonstrate anticancer activity through multiple mechanisms, including antioxidant activity, anti-inflammatory effects, cell cycle arrest, induction of apoptosis, inhibition of metastasis, anti-angiogenic activity, and modulation of key signaling pathways, such as PI3K/AKT/mTOR, MAPK/ERK, NF-κB, JAK/STAT, Wnt/β-catenin, p53, and epithelial–mesenchymal transition-related pathways. Most molecular and pathway-level evidence was obtained using isolated phenolic compounds in cell culture or animal models, whereas evidence directly examining whole EVOO consumption was largely observational and substantially more limited. Experimental studies also reported that oleocanthal induced lysosomal membrane permeabilization, whereas hydroxytyrosol and oleuropein promoted mitochondria-mediated apoptosis. Furthermore, preclinical combination studies suggested enhanced tumor-cell sensitivity to selected chemotherapeutic, targeted, and immunotherapeutic agents. However, these effects have not been established in patients. Human evidence remains limited mainly to observational dietary associations and small exploratory interventions, with no conclusive demonstration of cancer prevention or therapeutic efficacy. **Conclusions**: Isolated EVOO-derived phenolic compounds demonstrated promising anticancer mechanisms in preclinical models. However, these results should not be directly extrapolated to dietary EVOO because experimentally administered doses, bioavailability, metabolism, and food-matrix interactions differ substantially from human dietary exposure. Therefore, well-designed studies using chemically characterized EVOO, pharmacokinetic investigations, and controlled human trials are required before dietary or clinical recommendations can be made.

## 1. Introduction

Human health is a fundamental global concern due to the increasing burden of disease. Individual health and industrial development are strongly interconnected, directly or indirectly influencing the economic progress of a country [1]. Industrialization also poses several health hazards despite significant healthcare improvements, including exposure to environmental contaminants [2,3]. Furthermore, hectic lifestyles have reduced individuals’ involvement in cooking, leading to increased consumption of ready-to-eat and processed foods, which can disrupt lipid metabolism and significantly contribute to the development of cardiovascular disorders, myocardial infarction, and death [4,5,6]. Similarly, the physical and chemical properties of fats and oils deteriorate during overheating, causing oxidative rancidity, polymerization, isomerization, and loss of nutritional quality [7]. Lipid oxidation generates oxidative stress in the body, which disrupts normal cellular growth and development and may ultimately lead to the formation of cancerous cells. These abnormal cells proliferate and spread to other tissues and organs, resulting in metastasis [8,9]. Cancer is a malignant disease that can affect almost every organ of the body and is influenced by unhealthy environmental conditions, climatic variations, sedentary lifestyles, and poor dietary habits [10]. The global burden of cancer continues to rise, with approximately 20 million cases and around 9.7 million deaths reported worldwide [11,12].

Several metabolic alterations, including increased lactate production, enhanced lipogenesis, glutamine synthesis, reduced fatty acid oxidation, and aerobic glycolysis, contribute to cancer development and progression [13]. Additionally, dysregulation of multiple signaling pathways, such as cell cycle regulation, PI3K/AKT/mTOR, RAS/RAF/MEK/ERK, MAPK, Wnt/β-catenin, TGF-β/SMAD, p53, NF-κB, JAK/STAT, HIF-1α, and epithelial–mesenchymal transition (EMT), plays a critical role in tumorigenesis across various organs, including the liver, skin, brain, lungs, breast, cervix, uterus, and esophagus [14]. Various strategies, including pharmacological and dietary interventions, have therefore been investigated to reduce the prevalence of cancer. Extra virgin olive oil (EVOO), obtained from *Olea europaea*, is a principal component of the Mediterranean diet due to its health-promoting and disease-preventive properties [15]. EVOO consumption has been associated with several health outcomes, while its phenolic constituents have shown diverse biological activities in experimental studies, particularly related to cancer proliferation, inflammation, hypertension, cardiovascular diseases, obesity, cognitive disorders, and digestive complications. This review summarizes the anticancer potential of EVOO and EVOO-derived phenolics against various cancer types.

The novelty of this review lies in the synthesis of evidence published between 2015 and 2025, encompassing in vitro, in vivo, and epidemiological studies on the anticancer effects of EVOO. The present review extends previous reviews by providing an evidence-stratified and translational synthesis of the anticancer literature on EVOO. Rather than considering EVOO and olive-derived phenolics as interchangeable exposures, this review systematically distinguishes studies investigating whole EVOO, phenolic-enriched EVOO, olive phenolic extracts, isolated phenolic compounds, and oleic acid. Evidence is critically evaluated according to cancer type and level of evidence, allowing differences in the strength of preclinical and human evidence to be identified. In addition, this review integrates mechanistic findings with current knowledge regarding bioavailability, metabolism, achievable human exposure, and the limitations associated with translating pharmacological concentrations used in experimental studies into dietary or clinical settings. Finally, the review explicitly discusses the distinct biological roles of oleic acid and minor phenolic constituents, providing a framework for interpreting apparently conflicting findings reported in the literature. This evidence-based approach identifies current knowledge gaps and priorities for future mechanistic, pharmacokinetic, and clinical research.

Throughout this review, the term “EVOO” is used only when the investigated intervention involved consumption or administration of the intact extra virgin olive oil matrix. “Phenolic-enriched EVOO” and “olive phenolic extract” refer to preparations containing multiple olive-oil constituents, whereas hydroxytyrosol (HT), oleuropein, oleocanthal, and oleacein refer to the respective isolated compounds. Most pathway-level mechanistic evidence discussed in this review derives from isolated compounds tested at pharmacological concentrations in cell-culture or animal models. Such findings provide biological plausibility for the activities of EVOO-derived phenolics but should not be interpreted as direct evidence that dietary EVOO consumption produces equivalent anticancer effects. Extrapolation is limited by differences in dose, food-matrix interactions, interactions among constituents, gastrointestinal transformation, bioavailability, metabolism, and the concentrations achievable in human plasma and tissues. Accordingly, findings are attributed throughout the review to the specific preparation and experimental model investigated.

## 2. Materials and Methods

This narrative review was informed by a structured literature search of PubMed, Web of Science, ScienceDirect, and Google Scholar for English-language publications from 2015 to 2025. MeSH terms, Boolean operators (AND and OR), and specific keywords, such as extra virgin olive oil, EVOO, bioavailability, hydroxytyrosol, oleuropein, oleocanthal, oleacein, breast cancer, breast cancer and EVOO, breast cancer and hydroxytyrosol, breast cancer and oleuropein, breast cancer and oleocanthal, prostate cancer, prostate cancer and EVOO, prostate cancer and hydroxytyrosol, prostate cancer and oleuropein, prostate cancer and oleocanthal, colon cancer, colon cancer and EVOO, colon cancer and hydroxytyrosol, colon cancer and oleuropein, colon cancer and oleocanthal, pancreatic cancer, pancreatic cancer and EVOO, pancreatic cancer and hydroxytyrosol, pancreatic cancer and oleuropein, pancreatic cancer and oleocanthal, oral cancer, oral cancer and EVOO, oral cancer and hydroxytyrosol, oral cancer and oleuropein, oral cancer and oleocanthal, liver cancer, liver and EVOO, liver cancer and hydroxytyrosol, liver cancer and oleuropein, liver cancer and oleocanthal, brain cancer, brain cancer and EVOO, brain cancer and hydroxytyrosol, brain cancer and oleuropein, brain cancer and oleocanthal, blood cancer, blood cancer and EVOO, blood cancer and hydroxytyrosol, blood cancer and oleuropein, and blood cancer and oleocanthal, bone cancer and EVOO, bone cancer and hydroxytyrosol, bone cancer and oleuropein, and bone cancer and oleocanthal, gastric cancer and EVOO, gastric cancer and hydroxytyrosol, gastric cancer and oleuropein, and gastric cancer and oleocanthal, cervical cancer and EVOO, cervical cancer and hydroxytyrosol, cervical cancer and oleuropein, and cervical cancer and oleocanthal, thyroid cancer and EVOO, thyroid cancer and hydroxytyrosol, thyroid cancer and oleuropein, and thyroid cancer and oleocanthal, endometrial cancer and EVOO, endometrial cancer and hydroxytyrosol, endometrial cancer and oleuropein, and endometrial cancer and oleocanthal, ovarian cancer and EVOO, ovarian cancer and hydroxytyrosol, ovarian cancer and oleuropein, and ovarian cancer and oleocanthal, melanoma cancer and EVOO, melanoma cancer and hydroxytyrosol, melanoma cancer and oleuropein, and melanoma cancer and oleocanthal, were used to retrieve relevant studies.

In vitro, in vivo, and clinical studies related to these cancers that were accessible and published in English were included in this review. Studies published before 2015, inaccessible articles, and those published in non-English languages were excluded to ensure clarity and relevance. During evidence extraction and synthesis, studies were classified according to the investigated material—whole EVOO or olive oil, phenolic-enriched EVOO, multi-compound phenolic extract, or an isolated phenolic compound—and according to the level of evidence, including in vitro, animal, observational human, and interventional human studies. Conclusions were restricted to the material actually investigated. Results obtained using isolated compounds were not attributed to dietary EVOO consumption unless supported by corresponding whole-oil or human dietary evidence. Throughout the synthesis, mechanistic effects identified in cell-culture and animal studies were explicitly described as preclinical. Moreover, terms implying established prevention, treatment efficacy, therapeutic synergy, and protection of healthy tissues were avoided unless supported by controlled human evidence. Human observational findings were interpreted as associations, whereas biomarker changes in exploratory interventions were not considered evidence of tumor response or clinical benefit.

**Figure 1:**
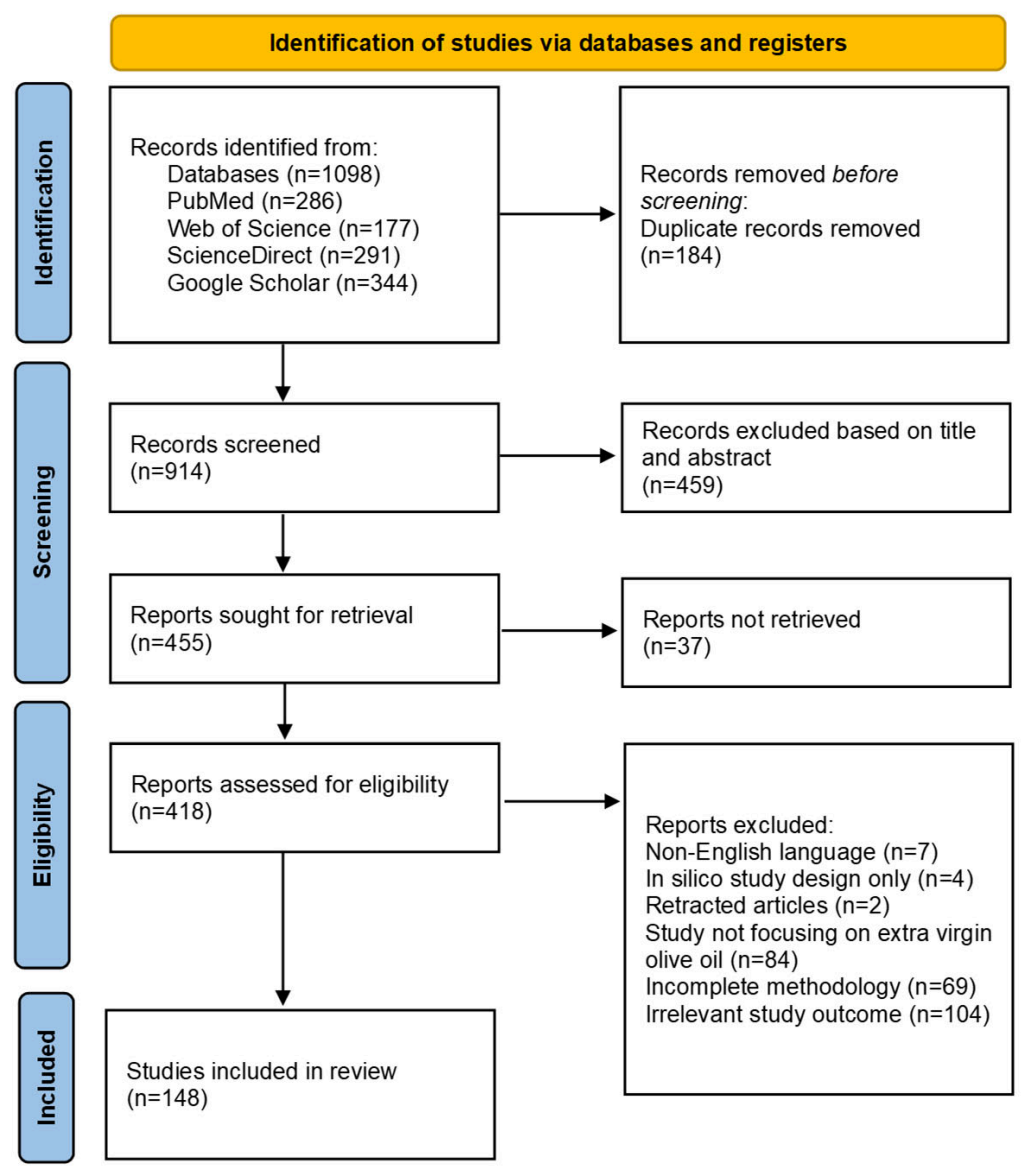
PRISMA flow diagram of the included studies.

The four online databases searched (PubMed, Web of Science, ScienceDirect, and Google Scholar) resulted in a total of 1098 records (PubMed, *n* = 286; Web of Science, *n* = 177; ScienceDirect, *n* = 291; Google Scholar, *n* = 344). After 184 duplicate studies were removed, 914 studies were screened by title and abstract, and 459 were excluded. Among the 455 reports sought for retrieval, 37 were not successfully retrieved. Therefore, 418 reports were eligible for full-text assessment. In this step, 270 reports were discarded for the following reasons: non-English language (*n* = 7), in silico study design only (*n* = 4), retracted articles (*n* = 2), studies not focusing on extra virgin olive oil (*n* = 84), incomplete methodology (*n* = 69), and irrelevant study outcomes (*n* = 104). Finally, 148 studies met all inclusion criteria and were included in the review (Figure 1).

## 3. Bioavailability

The bioavailability of foods, compounds, nutrients, and drugs is essential for their therapeutic activity within the body [16]. Bioactive compounds undergo various physicochemical and biochemical processes from ingestion to absorption, distribution, metabolism, and eventual elimination [17]. The bioavailability of compounds is influenced by multiple factors, including food composition, gastrointestinal tract functionality, pH, osmolality, viscosity, transit time, and gut microbiota [18]. Traditional and modern processing technologies, such as thermal treatment, milling, cooking, soaking, fermentation, germination, dehulling, and nanoencapsulation, help eliminate antinutritional compounds, including tannins, phytic acid, oxalates, saponins, lectins, and gossypol, thereby improving the bioavailability of nutrients and bioactive compounds [19].

The bioavailability of EVOO phenolic compounds is limited by various biochemical and enzymatic reactions occurring in the gastrointestinal tract [20]. Consequently, enhancing the bioavailability of EVOO bioactives remains a challenge for both researchers and the food and nutraceutical industry [21]. EVOO contains numerous bioactive compounds, including phenolic acids and flavonoids, which exert diverse biological effects in the human body [22]. For example, gastrointestinal secretions and digestive enzymes have minimal effects on the absorption of certain phenolic compounds, such as HT and tyrosol, allowing them to reach the intestine relatively intact [23]. In contrast, secoiridoids are relatively stable in the oral cavity after ingestion, but a significant proportion is degraded in the stomach, duodenum, and colon. Deglycosylation and cleavage of glycosidic linkages facilitate the absorption of secoiridoids in the small intestine, while oleacein is absorbed mainly through passive diffusion [24].

The absorption of phenolic compounds from EVOO is estimated to exceed 55–66 mol%. It has also been suggested that the consumption of approximately 50 g of olive oil per day provides around 2 mg of HT [25]. The bioaccessibility index of EVOO phenols ranges from 37% to 90%, indicating limited gastric absorption. Martin-Vertedor et al. reported that gastric and intestinal fluids reduce EVOO phenol bioaccessibility to approximately 60% and 90%, respectively [26].

**Figure 2:**
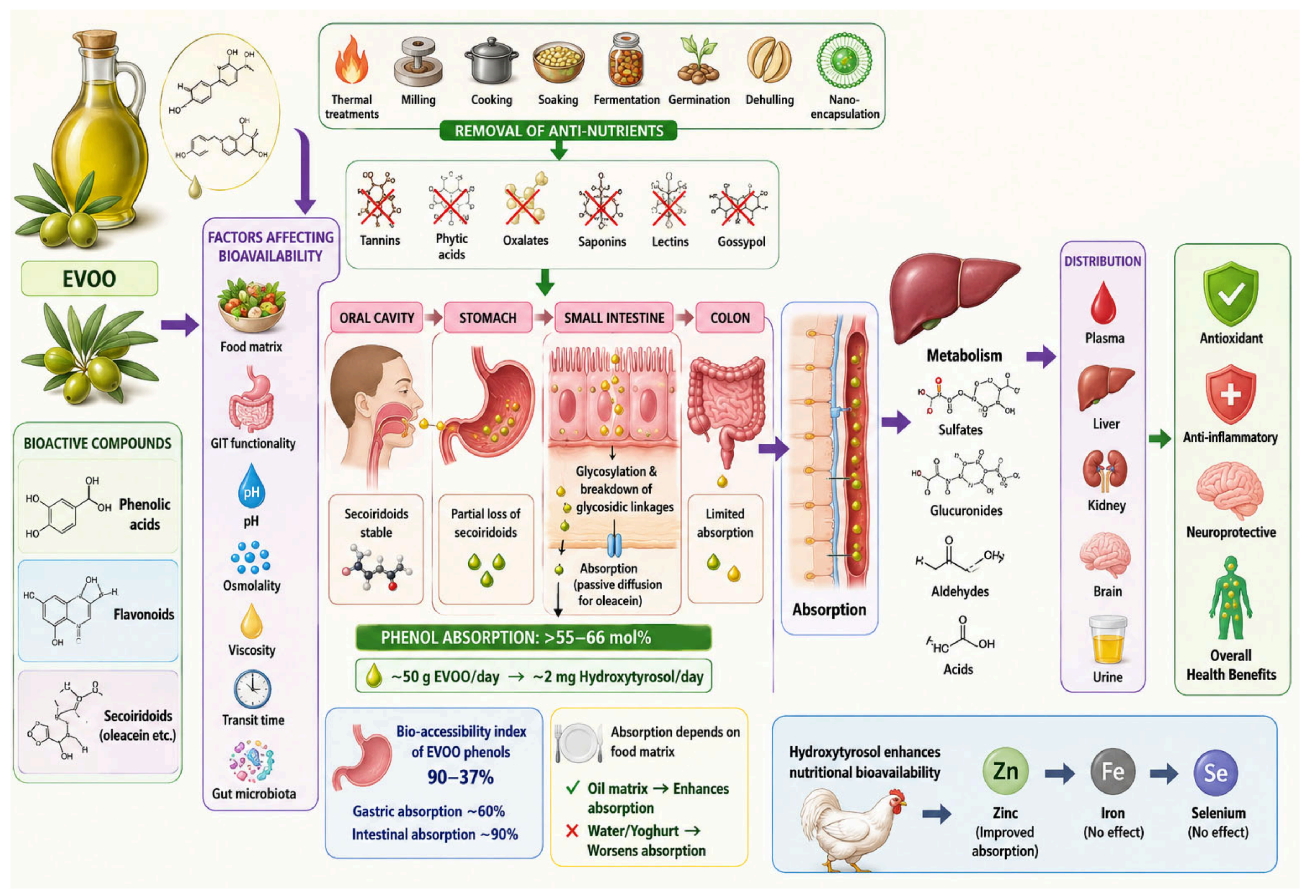
Bioavailability of extra virgin olive oil (EVOO) and its phenolic compounds, particularly through the interactions among the food matrix, gastrointestinal function, pH, osmolality, viscosity, transit time, and gut microbiota, and their effects on digestion, absorption, and bioaccessibility. Furthermore, the figure illustrates the mechanisms of antinutrient removal, absorption in the intestine, hepatic metabolism, and distribution in plasma and organs, and their associated therapeutic attributes.

The absorption of EVOO phenolics also depends on several factors, including the food matrix, the nature of the bioactive compounds, compound polarity, and gastrointestinal conditions [25]. Yerena et al. reported that EVOO phenolic absorption varies depending on the food matrix with which it is consumed; for example, an oil matrix enhances EVOO phenolic absorption, whereas water and yogurt may reduce its absorption [27]. After absorption, EVOO phenolic compounds are distributed and metabolized into various metabolites, including sulfates, aldehydes, acids, and glucuronides, which may contribute to their therapeutic effects, such as antioxidant, anti-inflammatory, and neuroprotective activities [28,29,30]. De las Hazas et al. investigated the association between HT intake and tissue uptake by supplementing rats with HT-enriched refined olive oil at doses of 1, 10, and 100 mg/kg [31]. Their results showed that HT accumulated in plasma, urine, liver, and brain tissues. Additionally, HT improved the nutritional bioavailability of certain food products by enhancing zinc absorption in broiler chicken meat, while showing no significant effect on iron and selenium absorption [32]. The bioavailability of EVOO phenolic compounds is illustrated in Figure 2.

The biological activity of virgin plant oils depends not only on their intrinsic phenolic composition but also on production practices, geographical origin, authenticity, and quality-control systems. A useful example is Morocco’s protected geographical indication for argan oil (“Huile d’Argane-Maroc”), where legal protection links the product to a defined geographical region, standardized production methods, and traceability requirements. The geographical indication system has been shown to improve product authenticity, preserve traditional processing techniques, and enhance quality assurance while simultaneously supporting biodiversity conservation, rural development, and women’s cooperatives [33]. These observations illustrate that the health-promoting properties of bioactive oils cannot be interpreted independently of their production system. Similar considerations are applicable to extra virgin olive oil, where cultivar, geographical origin, harvesting practices, processing conditions, storage, and certification influence the concentration and stability of phenolic compounds such as HT, oleuropein, oleacein, and oleocanthal, thereby affecting bioavailability and biological activity. Consequently, future clinical and mechanistic studies should report the chemical characterization and geographical origin of EVOO to improve reproducibility and facilitate comparison among studies.

## 4. Antioxidant Potential

Oxidative stress generated by free radicals, particularly reactive oxygen (ROS) and nitrogen species (RNS), is counteracted by antioxidants. Plants and their bioactive compounds, including phenolic acids, terpenoids, flavonoids, polyphenols, carotenoids, lignans, saponins, and phytosterols, exhibit antioxidant properties that help reduce oxidative stress and associated disorders, such as cardiovascular, respiratory, neurodegenerative, renal, hepatic, and reproductive diseases [34]. Plant-based oils, particularly EVOO, mitigate cellular and tissue damage by scavenging reactive species, and substantial evidence supporting these effects has been reported. Phenolic compounds present in EVOO, such as lignans, secoiridoids, luteolin, apigenin, hydroxybenzoic acid, ferulic acid, HT, and oleuropein, contribute significantly to its antioxidant potential and help reduce oxidative stress in the body [35,36].

## 5. Anticancer Perspectives

Evidence concerning EVOO and cancer originates from distinct types of exposure. Epidemiological studies generally assess olive oil intake, EVOO consumption, and Mediterranean dietary patterns, whereas most mechanistic studies use isolated compounds, such as HT, oleuropein, oleocanthal, and oleacein. The molecular effects discussed in the following sections were observed in cell culture and animal models and should be regarded as preclinical. These findings establish biological plausibility but do not demonstrate that dietary EVOO achieves equivalent exposures, prevents cancer, improves treatment response, or produces clinical benefit in humans. They identify potential mechanisms of EVOO-derived compounds but do not demonstrate that consumption of the intact EVOO matrix achieves the same doses or biological effects. Epidemiological associations reported in the following subsections should not be interpreted as evidence that EVOO independently prevents cancer or reduces cancer mortality.

Olive oil intake commonly occurs within a broader Mediterranean dietary and lifestyle pattern characterized by higher consumption of vegetables, fruits, legumes, whole grains, fish, and other potentially health-promoting foods. Individuals with higher olive oil intake may also differ in smoking, alcohol consumption, physical activity, body weight, socioeconomic status, screening participation, medication use, and access to healthcare. Although most observational studies adjust for several measured covariates, residual and unmeasured confounding, dietary measurement error, exposure misclassification, reverse causality, and selection bias cannot be excluded. Moreover, studies frequently assess total olive oil and Mediterranean diet adherence rather than chemically characterized EVOO, limiting attribution of the observed associations specifically to EVOO or its phenolic constituents.

EVOO is a complex food matrix whose major lipid fraction and minor phenolic fraction should not be considered biologically interchangeable. Oleic acid is the predominant monounsaturated fatty acid in EVOO, whereas HT, oleuropein, oleocanthal, and oleacein are minor phenolic constituents with distinct absorption, metabolism, and molecular targets. Most anticancer mechanisms reviewed here derive from isolated phenolics or phenolic-rich preparations. Findings obtained with isolated oleic acid or high-fat diets containing olive oil must therefore be interpreted separately and should not be attributed to EVOO phenolics or to customary dietary EVOO consumption.

Table 1 summarizes the principal studies discussed in this review according to the experimental or clinical model, investigated EVOO or olive-derived preparation, dose or concentration, molecular targets, main outcomes, and level of evidence. The table distinguishes whole EVOO and dietary interventions from phenolic-enriched preparations, extracts, and isolated compounds. Where a dose or concentration was not provided in the reviewed manuscript or source description, it is indicated as not reported.

As shown in Table 1, the depth of evidence differs substantially among cancer types. Breast and colorectal cancers have relatively broader preclinical evidence, including multiple cell models and cancer-specific animal studies; breast cancer additionally has limited human dietary evidence. Pancreatic cancer has an intermediate preclinical evidence base comprising cell, three-dimensional co-culture, and selected animal models, although direct human evidence remains indirect and limited. Moreover, gastric, oral, hematological, and brain cancers are supported by heterogeneous combinations of in vitro, limited animal, observational, and exploratory human evidence. By contrast, prostate and bone cancers rely predominantly on a small number of cell-based studies, with little or no cancer-specific human evidence. Liver cancer evidence is mainly observational, while direct mechanistic and interventional evidence is limited. Evidence for the cancers grouped under “Other Cancers” is exploratory and frequently derives from only one or two experimental studies. These categories should therefore not be interpreted as having equivalent evidential support, and none currently establishes the clinical efficacy of EVOO or its isolated phenolics.

The scope of the cancer-specific synthesis corresponds to the search strategy described in Section 2. Cancer types supported by sufficient site-specific literature are presented in dedicated subsections, whereas cervical, endometrial, ovarian, melanoma, non-melanoma skin, and thyroid cancers are grouped under “Other Cancers” because their evidence bases are comparatively limited and predominantly preclinical.

### 5.1. Breast Cancer

#### 5.1.1. Human and Dietary Evidence

Breast cancer is the most frequently diagnosed cancer among women worldwide, with approximately 2.3 million new cases and 0.67 million deaths reported annually [105]. Its occurrence is influenced by numerous reproductive, genetic, metabolic, environmental, and lifestyle-related factors [106]. A meta-analysis of observational studies reported that women with higher olive oil consumption, including intake levels of approximately ≥28 g/day in some analyses, had a relatively lower risk of breast cancer than women with lower consumption [75]. This finding represents an association and does not establish that olive oil or EVOO independently prevents breast cancer. The observed relationship may partly reflect the overall Mediterranean dietary pattern, differences in adiposity, physical activity, alcohol consumption, socioeconomic status, reproductive factors, use of hormone therapy, and participation in breast cancer screening. Furthermore, heterogeneity in dietary assessment and the frequent failure to distinguish total olive oil from chemically characterized EVOO limit compound-specific and causal interpretation.

#### 5.1.2. Preclinical Evidence

Phenolic compounds such as oleuropein and oleocanthal suppress breast cancer cell growth by inducing S-phase cell-cycle arrest and apoptosis through modulation of cyclin D1 and NF-κB, as well as downregulation of the CDK inhibitor p21 and growth factor-mediated HGF/Met-induced PI3K/AKT and MAPK/ERK pathways in MDA-MB-231 cells [37]. In addition, these compounds trigger caspase-dependent apoptosis by activating caspase-8 and caspase-3 and inducing PARP cleavage in triple-negative breast cancer (TNBC) cells through Met inhibition [38]. Moreover, the migration of MCF-7 and MDA-MB-231 tumor cells has been shown to decrease through the attenuation of PI3K/AKT/mTOR and NF-κB signaling pathways [39].

#### 5.1.3. In Vivo and Translational Interpretation

**Figure 3:**
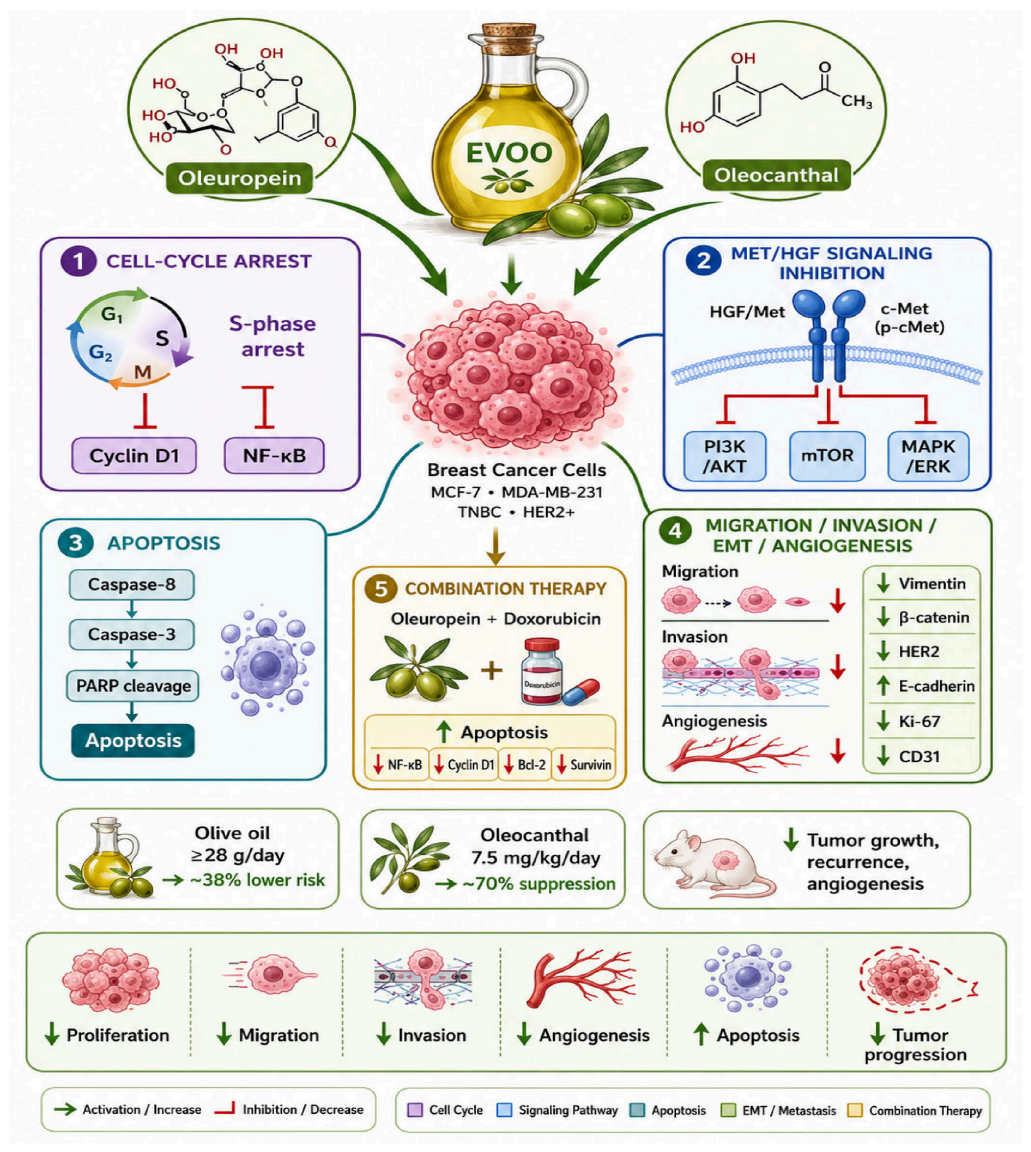
Breast cancer preventive mechanism of extra virgin olive oil, oleuropein and oleocanthal, which is due to their ability to arrest the cell cycle, inhibit the MET/HGF signaling pathway, induce apoptosis, and decrease migration, invasion, epithelial–mesenchymal transition, and angiogenesis.

Furthermore, in vivo findings support the results obtained from cell-based studies. Oral administration of oleocanthal at 7.5 mg/kg daily for 32 days reduced mammary tumor initiation and progression by 70% under the reported experimental conditions in the MMTV-PyVT transgenic mammary-tumor model [40]. Similarly, oleocanthal at 5 mg/kg inhibited the growth, proliferation, and invasion of MDA-MB-231 cells by reducing c-Met phosphorylation, Ki-67 expression, and CD31 levels [41]. In orthotopic breast cancer recurrence models, oleocanthal increased E-cadherin and decreased vimentin expression in BT-474 tumors and reduced phosphorylated HER2 and MET. In MDA-MB-231 recurrent tumors, oleocanthal reduced vimentin but did not restore E-cadherin expression [42]. Comparable findings were observed in the HER2^+^ model, where daily oleocanthal administration inhibited estrogen-driven growth of BT-474 tumors and reduced locoregional recurrence after surgery [43]. Additionally, oleuropein in combination with doxorubicin rendered MDA-MB-231 xenografts more apoptotic, accompanied by a significant reduction in NF-κB, cyclin D1, and anti-apoptotic proteins such as Bcl-2 and survivin compared with chemotherapy alone [44]. Collectively, isolated oleocanthal and oleuropein demonstrate antiproliferative, pro-apoptotic, anti-invasive, and anti-angiogenic activity in preclinical breast-cancer models. These findings provide biological plausibility for the activity of EVOO-derived phenolics but do not demonstrate that dietary EVOO prevents breast cancer or inhibits its progression, as illustrated in Figure 3. The epidemiological evidence remains associative and may be influenced by the overall dietary pattern and residual confounding, whereas the mechanistic evidence is predominantly compound-specific and preclinical.

### 5.2. Prostate Cancer

#### 5.2.1. Human and Dietary Evidence

Prostate cancer is the most prevalent cancer among men in both developed and developing countries and is strongly influenced by dietary habits and lifestyle practices. Preclinical studies indicate that isolated EVOO-derived phenolics, particularly HT, oleuropein, and oleocanthal, can modulate oncogenic pathways associated with prostate cancer, including PI3K/AKT, MAPK/ERK, NF-κB, and JAK/STAT signaling [9]. In vitro studies have shown that isolated HT, oleuropein, or oleocanthal can reduce tumor stem cell proliferation in prostate cancer cell lines, including LNCaP, DU145, 22Rv1, and PC 3. Oleuropein at high concentrations (100–500 μM) inhibited the proliferation of LNCaP and DU145 cells, primarily through Akt dephosphorylation [46]. Furthermore, HT demonstrated a dose-dependent cytotoxic effect on 22Rv1, LNCaP, and PC-3 cells, with IC_50_ values ranging from 9 to 41 μM, by promoting intrinsic apoptosis, as indicated by activation of caspase-3 and caspase-9 and PARP cleavage. Additionally, HT exhibited selective potency in eliminating hormone-sensitive (LNCaP) and castration-resistant (C4-2) cells while sparing normal prostate epithelial cells [9].

#### 5.2.2. Preclinical Evidence

In preclinical metastatic castration-resistant prostate-cancer models, oleocanthal attenuated tumor progression and recurrence through inhibition of the epigenetic regulator SMYD2 and its associated survival signaling [107]. These findings support further investigation of oleocanthal in prostate-cancer models but do not establish clinical activity or enhancement of androgen-deprivation therapy.

EVOO phenolics interfere with key molecular processes involved in cell survival and cell cycle progression. HT induces G_1_ phase arrest by downregulating cyclins D1/E and CDK2/4 and inhibiting CDK activity. This effect is associated with increased Bax expression and decreased levels of Bcl-2 and Bcl-xL [47]. HT also inhibits the phosphorylation of AKT and STAT3 and prevents the nuclear translocation of NF-κB in prostate cancer cells [48]. These molecular alterations lead to reduced AR signaling and PSA expression, thereby preventing androgen-driven cancer cell proliferation. Additionally, oleuropein enhances apoptotic signaling by increasing p53 and Bax expression while decreasing Bcl-2 and HIF-1α levels. These compounds target PI3K/AKT, MAPK/ERK, and NF-κB pathways, which are critical for prostate cancer progression [108,109].

#### 5.2.3. In Vivo and Translational Interpretation

The prostate-cancer evidence is derived predominantly from cell culture studies. Although animal studies in other tumor types have shown that HT and oleuropein can influence tumor growth and angiogenic markers, these findings do not constitute prostate-specific in vivo or clinical validation [9]. Inhibition of androgen-receptor, ERK, AKT, and PSA-related signaling suggests possible relevance to androgen-deprivation strategies [48,54,107]; however, this remains a preclinical hypothesis. Prostate-specific animal studies and controlled human trials are required before conclusions can be drawn regarding treatment enhancement and the management of castration-resistant disease.

### 5.3. Colon Cancer

#### 5.3.1. Human and Dietary Evidence

Colorectal cancer (CRC) is a common malignancy worldwide and ranks third among all cancer types. It is characterized by uncontrolled and abnormal proliferation of cells in the large intestine. According to global cancer statistics [105], approximately 1.93 million new cases and about 0.90 million deaths occur each year due to CRC. The management of colon cancer remains a global challenge, prompting researchers to explore innovative and effective therapeutic alternatives. The potential activity of EVOO-derived phenolics in colorectal cancer has been investigated primarily in preclinical models; direct evidence supporting their therapeutic use in patients is currently lacking.

#### 5.3.2. Preclinical Evidence

Experimental evidence includes studies of isolated EVOO-derived phenolics, phenolic extracts, and, less frequently, high-phenolic EVOO. In particular, isolated HT, oleuropein, and oleocanthal have been reported to reduce colon-cancer cell proliferation and invasion [79,110,111,112], suppress tumor growth [113], and induce apoptosis [114] in preclinical models. HT effectively inhibits the proliferation of colon cancer cells [50,51] by inducing G_2_/M phase cell-cycle arrest, inhibiting CDK1, degrading cyclin B1, and activating intrinsic apoptotic pathways [52]. Moreover, HT increases the expression of pro-apoptotic proteins such as caspase-3 and Bax, promoting caspase-dependent apoptosis in cultured CRC cells. HT also suppresses cancer cell proliferation and reduces EGFR expression in HT-29 colon cancer cells [53]. These in vitro findings were validated by in vivo studies, where HT-29 xenografts in mice treated with HT showed significantly reduced tumor growth and decreased EGFR levels [50,51]. Collectively, these studies suggest that HT inhibits the survival of CRC cells by altering redox homeostasis and regulating the EGFR/PI3K/Akt signaling pathway while promoting apoptosis.

#### 5.3.3. In Vivo and Translational Interpretation

Oleuropein, the primary secoiridoid present in olives, exhibits similar anticancer effects against colon cancer. Recent studies indicate that oleuropein acts as an antiproliferative and pro-apoptotic agent in CRC models [55]. It has been reported that oleuropein administration reduces proliferation in HT-29 cells by decreasing HIF-1α levels and enhancing p53 accumulation. This effect is associated with cell cycle alterations and increased DNA fragmentation, indicating mitochondria-mediated apoptosis [56]. Furthermore, oleuropein inhibits inflammatory and proliferative pathways, including NF-κB, AKT, COX-2, and Wnt/β catenin signaling in colon cancer cells, thereby demonstrating chemopreventive potential [54,57].

Oleocanthal has also demonstrated inhibitory activity in preclinical colorectal-cancer models. A recent study evaluating the anticancer activity of EVOO phenolics reported that olive polyphenols, particularly oleocanthal, exhibited strong inhibitory activity against CRC (COLO-320DM) cell proliferation, with an IC_50_ value of 9.8 µM [58]. Moreover, dietary interventions using food-grade oleocanthal or high phenolic EVOO in mouse xenograft models markedly reduced colon tumor burden (>70%) and significantly decreased local recurrence, with relapse inhibition exceeding 85% [59]. Overall, isolated EVOO-derived phenolics and selected high-phenolic preparations have shown antiproliferative, pro-apoptotic, and tumor-suppressive activity in cell and animal models. These findings do not establish that customary dietary EVOO prevents or treats colorectal cancer in humans.

### 5.4. Pancreatic Cancer

#### 5.4.1. Human and Dietary Evidence

Pancreatic cancer is an aggressive malignancy of the pancreas that is often diagnosed at advanced stages due to mild early clinical symptoms, resulting in poor prognosis and high mortality rates [115]. Major risk factors include smoking, obesity, diabetes, pancreatitis, and other metabolic disorders, contributing to approximately 0.49 million new cases and 0.46 million deaths worldwide according to the International Agency for Research on Cancer [105]. Lifestyle and dietary patterns have been investigated in relation to pancreatic cancer risk [116]. However, evidence specifically concerning EVOO remains limited because most epidemiological studies evaluate adherence to the Mediterranean diet as a composite exposure rather than EVOO independently. EVOO is one component of this dietary pattern, alongside vegetables, fruits, legumes, whole grains, fish, and relatively low consumption of processed foods and red meat. Consequently, associations involving Mediterranean-diet adherence cannot be attributed specifically to EVOO or its phenolic compounds.

#### 5.4.2. Preclinical Evidence

A 2023 meta-analysis of eight cohort studies involving approximately 1.3 million participants reported that stronger adherence to the Mediterranean diet was associated with an approximately 18% lower relative incidence of pancreatic cancer [117]. This estimate should not be interpreted as demonstrating an 18% causal reduction produced by EVOO. Residual confounding by smoking, obesity, diabetes, alcohol consumption, physical activity, socioeconomic status, and other dietary components remains possible. Self-reported dietary assessment, variation in Mediterranean diet scoring systems, and changes in diet during follow-up may also contribute to measurement error and between-study heterogeneity.

#### 5.4.3. In Vivo and Translational Interpretation

Several in vitro studies have examined the effects of major EVOO phenolics on pancreatic cancer cells. Oleuropein and HT inhibit pancreatic ductal adenocarcinoma (PDAC) cell proliferation and induce apoptosis at µM concentrations. Treatment of human MIA PaCa-2 cells with oleuropein or HT for 72 h significantly reduced cell viability (IC_50_ ≈ 150 μM and 75 μM, respectively) and induced G_2_-phase arrest, ultimately leading to caspase-3-dependent apoptosis [46]. This effect was associated with increased expression of the AP-1 transcription factors c-Jun and c-Fos, highlighting the role of stress-induced signaling in apoptosis. HT treatment of PANC-1 cells also produced a dose-dependent decrease in viability. Apoptosis was observed at concentrations as low as 19 μM and was accompanied by increased caspase-9 and Bax expression and reduced MMP-2/9 expression. Additionally, HT suppressed STAT3 and cyclin D1 expression in Panc02 (murine) cells, thereby inhibiting cell growth [60].

Oleocanthal, another phenolic compound found in EVOO, acts through lysosomal and stromal mechanisms. Oleocanthal induces lysosomal membrane permeabilization in PDAC cell models, leading to the release of cathepsins into the cytosol and rapid cytotoxicity in cancer cells while sparing normal cells. This LMP effect is attributed to the inherent fragility of cancer cell lysosomes. Oleocanthal exhibits stronger anticancer activity than oleuropein or HT due to its unique dialdehyde structure and lysosomotropic properties [118].

Only a limited number of animal studies have investigated the effects of EVOO phenolics on pancreatic tumors. One study reported that HT enhances the efficacy of anti-CD47 immunotherapy in mice with pancreatic ductal adenocarcinoma, suggesting that HT can modulate the immune microenvironment to suppress PDAC progression [119]. Similarly, oleocanthal has shown promising results in a pancreatic neuroendocrine tumor model, where it significantly inhibited tumorigenesis and improved survival in a transgenic PNET-prone mouse strain [61,62].

Preclinical evidence indicates that isolated HT, oleuropein, and oleocanthal may have chemopreventive or adjuvant potential in experimental pancreatic cancer models. These compounds can sensitize tumor cells to treatment by inhibiting cell-cycle progression and survival pathways such as STAT3 and c-Met while promoting apoptosis. Oleuropein and HT may activate AP-1 transcription factors (c-Jun/c-Fos), thereby enhancing the sensitivity of PDAC cells to gemcitabine-induced apoptosis or modulating downstream signaling pathways such as Bim [46]. Additionally, HT may influence the tumor microenvironment by modulating immune responses and cytokine production [119]. Its immunomodulatory effects, including reduced myeloid-derived suppressor cells and increased M1 macrophages, suggest potential synergy with immunotherapies. Indeed, HT combined with anti-CD47 antibodies significantly enhanced antitumor efficacy in vivo [60]. Furthermore, EVOO phenolics used in combination with chemotherapy may induce oxidative stress and modulate inflammasome signaling to overcome PDAC resistance [120].

**Figure 4:**
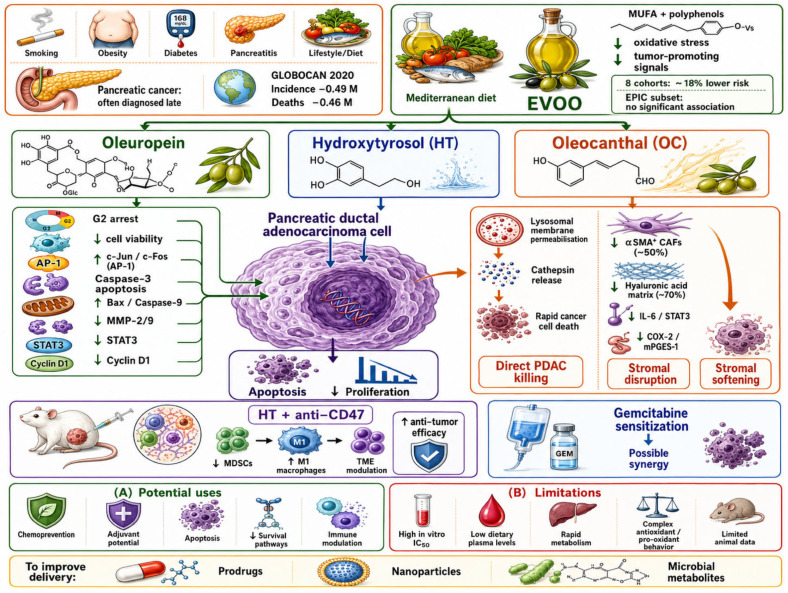
Therapeutic properties of extra virgin olive oil (EVOO) and its phytochemicals in pancreatic cancer, particularly focusing on their ability to inhibit proliferation, trigger apoptosis, inhibit tumor stroma, modify the tumor microenvironment, and enhance the response to other therapies, such as gemcitabine and anti-CD47 therapy.

Despite these promising findings, several challenges remain. The in vitro IC_50_ values of oleuropein and HT (ranging from tens to hundreds of μM) are considerably higher than the plasma concentrations achievable through dietary intake. Additionally, these compounds exhibit complex pharmacological properties, acting as antioxidants or pro-oxidants depending on the biological context, which complicates dose optimization and clinical application. Rapid metabolism may also explain the limited bioavailability of certain phenolics. Current research is therefore exploring strategies to enhance in vivo activity, including esterified prodrugs, nanoparticle delivery systems, and microbiota-derived metabolites [54,121]. The pancreatic-cancer evidence reviewed above is predominantly compound-specific and preclinical. It should therefore not be interpreted as evidence that dietary EVOO consumption achieves equivalent systemic concentrations or therapeutic effects. The reported pancreatic-cancer mechanisms of isolated EVOO-derived phenolics are summarized in Figure 4.

Minor EVOO biophenols, such as tyrosol and oleacein, also exhibit anticancer properties across various cancer types; however, information regarding their effectiveness in pancreatic cancer models remains limited. In summary, EVOO phenolics appear to interfere with key mechanisms involved in pancreatic tumor progression, including the induction of oxidative stress and apoptosis, cell-cycle arrest, and inhibition of proliferative signaling pathways, while exerting minimal effects on non-tumor cells. The in vitro and in vivo mechanisms of EVOO phenolics against pancreatic carcinoma are summarized in Table 2.

### 5.5. Bone Cancer

#### 5.5.1. Human and Dietary Evidence

Recent literature has revealed that phenolic compounds found in EVOO, such as oleuropein, HT, and oleocanthal, can inhibit the differentiation and survival of bone cancer cells.

#### 5.5.2. Preclinical Evidence

Oleuropein reduced the proliferation of MG-63 osteosarcoma cells and induced G_0_/G_1_-phase cell-cycle arrest, with a reported IC_50_ of approximately 22 µg/mL [64]. A separate MG-63 study evaluated oleuropein alone and in combination with adriamycin and reported greater cytotoxic activity with the combination than with either treatment alone, together with changes in cell survival and death-related responses [63]. These findings suggest that oleuropein may influence osteosarcoma cell proliferation and sensitivity to chemotherapy under in vitro conditions.

#### 5.5.3. In Vivo and Translational Interpretation

Overall, the bone-cancer evidence is limited to a small number of osteosarcoma studies, principally involving oleuropein and HT [63,64,122,123]. These studies report cell-cycle arrest, autophagy, apoptosis, and possible interactions with chemotherapeutic agents in experimental models. However, bone-cancer-specific primary evidence for oleocanthal remains insufficient, and the broader mechanisms reported in other cancer types should not be attributed directly to osteosarcoma. These in vitro and animal findings suggest that specific isolated EVOO-derived phenolics may have chemopreventive or adjunctive potential against bone cancers; however, they do not provide evidence for an effect of dietary EVOO consumption. Moreover, the combination of oleuropein with chemotherapeutic drugs demonstrates enhanced cytotoxicity in cellular models [63]. Overall, EVOO phenolics, including oleuropein, HT, and oleocanthal, reduce the survival of osteosarcoma cells by inducing apoptosis, causing cell cycle arrest, and promoting autophagy [64,124].

### 5.6. Oral Cancer

#### 5.6.1. Human and Dietary Evidence

Direct epidemiological evidence concerning EVOO intake and oral cancer is limited. A systematic review and meta-analysis of predominantly case–control studies reported that stronger adherence to the Mediterranean diet was associated with lower risks of head, neck, and oral cavity cancers [70]. Additional observational studies have reported inverse associations between dietary patterns rich in fruits, vegetables, and olive oil and oral cancer occurrence, whereas pro-inflammatory dietary patterns rich in red and fried meats have been associated with higher risk estimates [71,72]. These findings do not establish an independent protective effect of EVOO because olive oil intake is closely correlated with multiple other components of the Mediterranean diet. Moreover, case–control evidence is particularly susceptible to recall bias, selection bias, and changes in diet following early symptoms. Residual confounding by tobacco use, alcohol consumption, human papillomavirus infection, oral hygiene, socioeconomic status, and access to dental care also limits causal interpretation.

#### 5.6.2. Preclinical Evidence

Several investigations have demonstrated that EVOO polyphenols inhibit the progression of oral epithelial carcinoma and induce apoptosis. HT and oleuropein suppressed tumor cell growth in human oral cancer (KB) cells and human tongue cancer (HNO-97) cells by inducing G_0_/G_1_-phase cell-cycle arrest and inhibiting wound closure in scratch assays [65]. Moreover, oleocanthal has been associated with cancer cell death through lysosomal membrane permeabilization [54]. These in vitro findings demonstrate significant inhibition of oral cancer cell progression through enhanced apoptotic induction and cell-cycle arrest [66]. In addition, gross and histological analyses in animal studies have shown a reduction in tongue lesions following treatment with olive-derived phenolics [69]. These preclinical findings suggest that olive phenolics may impede the initiation and progression of oral cancer. These compounds exert anticancer effects through multiple signaling pathways by modulating intercellular communication and gene expression, as well as inhibiting the NF-κB pathway and pro-inflammatory cytokines, particularly COX-2 [71]. HT selectively increases pro-apoptotic protein levels (BAX) while decreasing anti-apoptotic protein expression in oral cancer cells [67]. Similarly, oleuropein promotes G_1_-phase cell-cycle arrest by downregulating cyclins and cyclin-dependent kinases (CDKs) and activating caspases [68]. These actions induce oxidative stress in tumor cells and inhibit proliferative signaling pathways such as PI3K/Akt and MAPK, while also suppressing angiogenesis and metastasis [75,125]. The combined effects of cell-cycle arrest and reduced inflammation contribute to tumor suppression by promoting apoptosis and slowing cancer cell proliferation [76].

#### 5.6.3. In Vivo and Translational Interpretation

**Figure 5:**
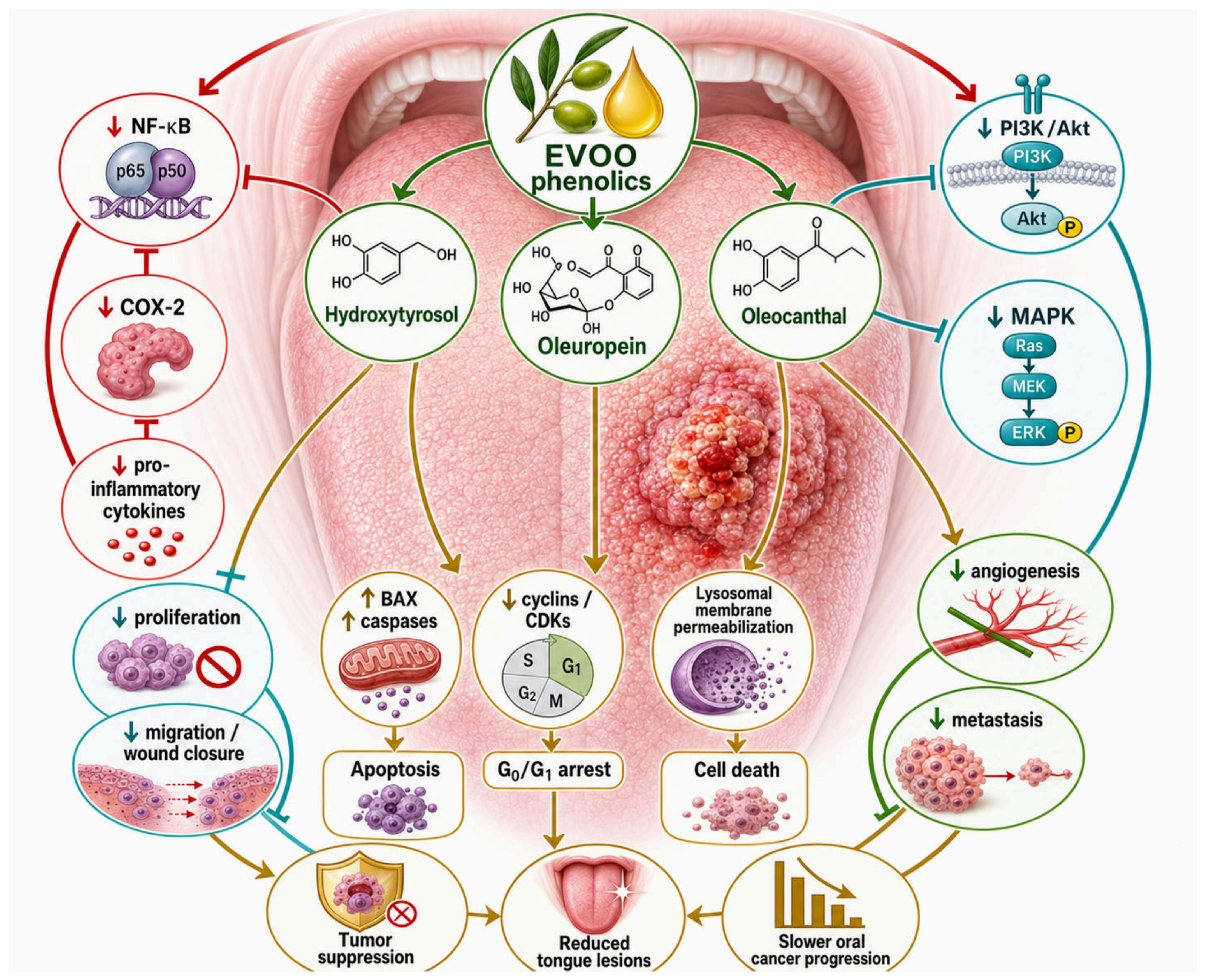
Oral tongue cancer-inhibiting properties of EVOO phenolics, particularly hydroxytyrosol, oleuropein and oleocanthal. The figure illustrates their ability to inhibit inflammatory pathways, including NF-κB, COX-2, PI3K/Akt and MAPK, as well as to inhibit proliferation, migration, angiogenesis, and metastasis.

Overall, isolated HT, oleuropein, and oleocanthal demonstrate potentially relevant biological activity in preclinical oral cancer models. Epidemiological studies of Mediterranean dietary patterns provide supportive but non-causal associations and do not establish an independent effect of EVOO. Therefore, neither the observational findings nor the compound-specific experimental results demonstrate that customary dietary EVOO intake prevents or treats oral cancer. In cultured tongue squamous-cell carcinoma cells, oleuropein increased the cytotoxic effect of 5-fluorouracil under the experimental conditions [66]. Moreover, controlled animal studies and human trials are required before recommendations can be made [126,127]. Figure 5 summarizes the anticancer mechanisms reported for isolated EVOO-derived phenolics in experimental oral cancer models.

### 5.7. Liver Cancer

#### 5.7.1. Human and Dietary Evidence

Liver cancer, predominantly hepatocellular carcinoma (HCC), is a major contributor to global cancer mortality. Chronic viral hepatitis, nonalcoholic fatty liver disease (NAFLD), and metabolic syndrome are among the principal risk factors [128]. Observational studies have examined olive oil consumption and Mediterranean dietary patterns in relation to HCC. An Italian case–control study and a pooled analysis reported that participants with higher olive oil intake had lower relative estimates of HCC risk than those with lower intake [129].

#### 5.7.2. Preclinical Evidence

A 2024 meta-analysis of cohort and case–control studies reported that healthier dietary patterns, including the Mediterranean diet, were associated with approximately 35–50% lower relative estimates of HCC risk [130]. Another systematic review and meta-analysis found an inverse association between Mediterranean diet adherence and liver cancer incidence [73]. These estimates describe differences observed between dietary-exposure groups and should not be interpreted as demonstrating that EVOO caused a corresponding reduction in HCC incidence. Furthermore, case–control studies conducted in Mediterranean populations suggest that daily consumption of approximately 20 g of EVOO may be associated with reduced HCC incidence by nearly 50% compared with minimal intake, potentially through modulation of lipid metabolism and oxidative stress [74]. Prospective evidence has been inconsistent. For example, analyses from the EPIC cohort did not identify a significant association in some non-Mediterranean populations [131]. Differences in background diet, olive-oil type, intake range, duration of follow-up, population characteristics, and adjustment for major HCC risk factors may contribute to this inconsistency. Therefore, the current epidemiological evidence supports a hypothesis-generating inverse association but does not establish that EVOO independently prevents HCC. Residual confounding and unmeasured bias remain possible even after multivariable adjustment.

### 5.8. Gastric Cancer

#### 5.8.1. Human and Dietary Evidence

Gastric adenocarcinoma is primarily associated with chronic inflammation induced by Helicobacter pylori infection, as well as dietary and environmental factors [46]. Bioactive compounds derived from medicinal plants, particularly oleuropein, HT, and oleocanthal, demonstrate gastroprotective and anticancer effects by modulating several signaling pathways, including PI3K/Akt/mTOR, NF-κB, Wnt/β-catenin, p53, and COX-2-related pathways, as well as suppressing oncogenic receptors such as EGFR and c-Met [77].

#### 5.8.2. Preclinical Evidence

Studies have shown that oleocanthal-containing EVOO extracts increase intracellular ROS levels and activate p53 signaling in gastric cancer cells, thereby influencing cell-cycle regulation [76]. Furthermore, oleuropein (50–500 μM) reduces the viability of human gastric adenocarcinoma cells and induces apoptosis in a dose- and time-dependent manner. It also modulates the expression of pro- and anti-apoptotic genes, including increased Bax expression, activation of caspases, and decreased Bcl-2 levels [54,75]. Similar effects have been reported for HT, oleocanthal, and other EVOO phenolics, which commonly induce cell-cycle arrest followed by caspase-dependent mitochondrial apoptosis. Collectively, EVOO phenolics activate multiple tumor-suppressive mechanisms by elevating oxidative stress in tumor cells, reactivating p53 signaling, and inhibiting survival pathways, ultimately suppressing gastric cancer cell growth [9,78]. These mechanistic findings derive primarily from isolated compounds and should be distinguished from observational associations involving dietary EVOO and olive-oil intake.

Epidemiological evidence has suggested an inverse association between olive oil or EVOO intake and gastrointestinal cancer outcomes. For example, an Italian cohort reported lower gastrointestinal cancer mortality estimates among participants with higher EVOO consumption [74]. The reported relative difference of approximately 50–60% should not be interpreted as demonstrating that EVOO caused an equivalent reduction in gastric cancer mortality. The outcome included gastrointestinal cancers rather than gastric cancer alone, and the observational design cannot exclude residual confounding by smoking, alcohol consumption, Helicobacter pylori infection, body weight, socioeconomic status, healthcare use, and the overall Mediterranean dietary pattern. Reverse causality is also possible if participants with pre-existing illness or early symptoms modified their dietary intake. Accordingly, this study provides associative evidence that requires confirmation in prospective analyses with repeated dietary assessment and more cancer-specific outcomes.

#### 5.8.3. In Vivo and Translational Interpretation

Although in vivo evidence remains limited, several experimental models support the cytotoxic effect of EVOO in gastric pathology. In *H. pylori-*infected mice, dietary supplementation with EVOO significantly reduced the development of gastric mucosal lesions. EVOO also demonstrated in vitro anti-*H. pylori* activity with a minimum inhibitory concentration of approximately 230 μg/mL [80]. In these experimental models, EVOO supplementation reduced ulcer indices and restored near-normal gastric histology compared with untreated controls. Histological analysis further confirmed that the gastric mucosa of *H. pylori-*infected mice receiving EVOO supplementation closely resembled that of healthy animals.

Phenolic compounds present in olive oil have demonstrated activity against multiple *H. pylori* strains, including antibiotic-resistant variants. These phenolics can inhibit bacterial adhesion and protect gastric epithelial cells from DNA damage induced by infection, suggesting a potential role in preventing bacteria-associated carcinogenesis. Although large animal xenograft studies are still lacking, the observed anti-inflammatory and mucosal-protective effects provide promising indications of chemopreventive potential [81].

Isolated EVOO-derived phenolics and selected phenolic extracts act on multiple chemopreventive targets in experimental gastric cancer models. Oleocanthal-enriched EVOO extracts stimulate ROS production in gastric cancer cells and enhance the cytotoxic effects of conventional chemotherapeutic agents, thereby increasing treatment efficacy [78]. Olive oil polyphenols, including oleocanthal, oleuropein, HT, and oleacein*,* collectively regulate key cancer-related pathways by inducing apoptosis, inhibiting PI3K/Akt and MAPK signaling, suppressing angiogenic factors, and reducing inflammatory mediators such as COX-2 and IL-8 [54].

Experimental studies have shown that methanolic extracts from Greek table olives reduce AGS gastric cancer cell proliferation, promote apoptosis, and decrease ICAM-1 and IL-8 expression, highlighting their antiproliferative and anti-inflammatory properties. Furthermore, when combined with chemotherapeutic drugs, such as cisplatin, tamoxifen, and doxorubicin, olive oil polyphenols enhance anticancer efficacy [79]. EVOO phenolics also exhibit anti-adhesion activity against *H. pylori*, thereby protecting gastric epithelial cells from infection-induced DNA damage and inflammation. In normal gastric cells, these phenolic extracts maintain antioxidant capacity and metabolic balance, suggesting lower toxicity toward the tested nonmalignant cell line under the experimental conditions [132].

Epidemiological studies further support a protective association between dietary polyphenols and gastric cancer risk. A meta-analysis reported a 29% reduction in gastric cancer risk among individuals with high polyphenol intake, with a stronger protective effect observed in females [133]. Overall, in vitro and animal studies demonstrate that EVOO phenolics induce apoptosis, promote G_1_-phase cell-cycle arrest, generate selective oxidative stress in tumor cells, and enhance the effectiveness of chemotherapeutic treatments while sparing normal cells. These findings highlight the potential of EVOO phenolics as preventive or adjunctive therapeutic agents for gastric cancer and warrant further clinical investigation [76,134,135].

### 5.9. Blood Cancer

#### 5.9.1. Human and Dietary Evidence

Numerous studies demonstrate that EVOO phenolics trigger programmed cell death and inhibit the growth of leukemia, lymphoma, and myeloma cells. A phenolic extract from EVOO significantly reduced the proliferation of HL60 (acute promyelocytic leukemia) cells and induced apoptosis. Similarly, HT arrested HL60 cells in the G_0_/G_1_ phase and promoted cytochrome c release and caspase 3 activation [82]. In K562 (chronic myeloid leukemia, CML) cells, oleuropein (the secoiridoid precursor of HT) reduced cell viability in a time- and dose-dependent manner (IC_50_ ≈ 244 µM at 72 h) while also disturbing cellular redox balance [84].

#### 5.9.2. Preclinical Evidence

Oleocanthal also exerts significant effects on hematopoietic cancer cells. In a panel of tumor cell lines (Jurkat, CEM, Raji, and K562), OLC induced sub-G_1_ accumulation and Annexin V staining, indicating apoptosis. Importantly, non-malignant cell lines showed substantial resistance to oleocanthal, suggesting selective toxicity toward cancer cells [85]. Across multiple studies, key apoptotic mediators such as caspase 3, caspase 8, and caspase 9 were activated. For example, oleocanthal induced mitochondrial depolarization and increased ROS production, whereas HT promoted cytochrome c release [82]. However, complete inhibition of caspases did not fully prevent oleocanthal-induced cell death, indicating that both caspase-dependent and caspase-independent mechanisms may be involved. In contrast, Gulbay and colleagues reported that oleuropein reduced K562 cell viability without significant changes in caspase 3 activity, suggesting the involvement of alternative cell death pathways such as oxidative stress-mediated mechanisms [84]. Overall, EVOO phenolics consistently induce apoptosis in blood cancer cells, largely through mitochondrial damage, increased ROS production, and DNA fragmentation.

#### 5.9.3. In Vivo and Translational Interpretation

EVOO-derived phenolics also altered cell-cycle regulation in several hematological cancer cell models. HT increased G_0_/G_1_-phase accumulation and modified CDK6, p21, and p27 expression in HL-60 acute promyelocytic leukemia cells and affected cell-cycle distribution in Jurkat T-cell acute lymphoblastic leukemia cells (Table 3) [83]. Jurkat cells should therefore be classified as a T-cell acute lymphoblastic leukemia model rather than a chronic lymphocytic leukemia model. In K562 chronic myeloid leukemia cells, oleuropein induced sequential G_0_/G_1_- and G_2_/M-phase arrest accompanied by altered cyclin and cyclin-dependent kinase-inhibitor expression [84]. These findings are derived from in vitro models and should be distinguished from the separate pilot intervention in patients with early-stage chronic lymphocytic leukemia. In the human chronic lymphocytic leukemia (CLL) intervention, participants consumed chemically characterized high-phenolic EVOO containing oleocanthal and oleacein, and changes in p21, cyclin D, apoptotic biomarkers, and circulating lymphocyte counts were reported [87]. These clinical biomarker observations were obtained in patients and should not be described as findings from Jurkat cells.

Oleocanthal consistently induces oxidative stress in malignant hematopoietic cells. In several tumor cell lines, OLC triggered mitochondrial outer membrane depolarization (Δψm) and activated the intrinsic apoptotic cascade [85]. An oleocanthal-enriched EVOO fraction (OCF) also caused a substantial increase in intracellular ROS levels and induced cell cycle arrest in gastric cancer cells, indicating that ROS generation is a key feature of OLC activity. Furthermore, OLC significantly reduced phosphorylation of Akt and ERK1/2 in multiple myeloma cells, thereby suppressing two major survival pathways [78]. It also inhibited NF-κB and STAT3 signaling in non-hematopoietic tumor models, suggesting that similar mechanisms may occur in leukemia and lymphoma cells [136].

HT exhibits a dual redox character. While its catechol structure confers strong antioxidant activity (scavenging O_2_^−^, HOCl, and ROO·), several studies have reported HT-induced hydrogen peroxide (H_2_O_2_) production, release of labile iron, and activation of downstream JNK/p38 signaling pathways in myeloid cells [137]. This shift toward pro-oxidant activity can disrupt cellular redox balance and promote apoptosis. Oleuropein also exhibits a net pro-oxidant effect. In chronic myeloid leukemia K562 cells, oleuropein decreased both total oxidant and antioxidant levels while increasing the oxidative stress index, indicating disruption of redox homeostasis in favor of cell death pathways [46]. Collectively, EVOO phenolics converge on oxidative signaling mechanisms, characterized by ROS generation and mitochondrial depolarization, along with inhibition of pro-survival pathways such as PI3K/Akt, ERK/MAPK, NF-κB, and STAT3, thereby promoting apoptosis in leukemia and lymphoma cells.

EVOO phenolics may also influence epigenetic regulation and differentiation pathways in hematologic malignancies [138]. In multiple myeloma cell lines, oleacein has been shown to decrease the transcription factor Sp1, leading to reduced expression of HDAC1, HDAC2, HDAC3, HDAC4, and HDAC6 and a corresponding increase in histone acetylation [86]. Oleacein also enhanced the sensitivity of multiple myeloma cells to the proteasome inhibitor carfilzomib in vitro [86]. These results directly demonstrate a role for oleacein in the regulation of the Sp1/HDAC axis in vitro. However, these effects have not yet been confirmed in animal models or humans.

Oleocanthal has also been independently reported to induce depolarization of mitochondria in both leukemia and lymphoma cell models, to induce the production of ROS, and to inhibit the phosphorylation of AKT and ERK, while also activating caspases [85]. Collectively, these compound-specific observations point to the possibility that olive-derived phenolics may impact both survival and epigenetic pathways in hematological cancer cells. However, clinical relevance has yet to be determined.

Information regarding the effects of EVOO phenolics in xenograft trials of hematological malignancies, such as leukemia or lymphoma, remains limited. Most of the available in vivo data originate from a human dietary intervention study. In a pilot randomized trial, patients with early-stage CLL consumed 40 mL/day of EVOO enriched with oleocanthal and oleacein for six months [87]. Compared with baseline values and a low-phenol control oil, the high-phenol EVOO significantly reduced lymphocyte and white blood cell counts while increasing apoptosis markers (cytokeratin-18 fragment ccK18 and Apo1/Fas) and the cell-cycle inhibitor p21. In contrast, the levels of anti-apoptotic proteins such as survivin and cyclin D decreased. These biomarker changes are consistent with altered apoptotic and cell-cycle signaling but do not demonstrate objective tumor response, delayed disease progression, or improved survival [85]. The pilot findings suggest that high-phenolic EVOO may influence selected hematological biomarkers in early-stage chronic lymphocytic leukemia; however, the small sample size and exploratory outcomes preclude conclusions regarding clinical efficacy [87].

In vitro evidence indicates that EVOO phenolics target pathways involved in the survival of blood cancer cells. These compounds inhibit pro-survival signaling by activating caspases and suppressing Akt/ERK/STAT3 pathways. For example, oleacein significantly enhances the efficacy of the proteasome inhibitor carfilzomib in multiple myeloma cells. The combined treatment leads to greater downregulation of histone deacetylases (HDACs), increased histone acetylation, and enhanced apoptosis compared with individual treatments [86]. Although no clinical trials have yet examined polyphenol–chemotherapy combinations in leukemia, preclinical studies in solid tumors suggest improved therapeutic efficacy, such as the synergistic effect of oleuropein with doxorubicin [46]. Importantly, normal hematopoietic cells appear less susceptible to these compounds, while oleocanthal selectively eliminated leukemic cell lines while sparing non-tumor cells [85]. Overall, EVOO polyphenols may enhance the sensitivity of cancer cells to treatment and suppress oncogenic signaling pathways while exhibiting minimal toxicity toward normal cells. Evidence regarding hematological malignancies is presently limited to EVOO phenolic extracts and isolated compounds investigated in in vitro and preclinical models. Therefore, no conclusion can be drawn regarding the prevention or treatment of blood cancers through dietary EVOO consumption.

### 5.10. Brain Cancer

#### 5.10.1. Human and Dietary Evidence

Glioblastoma (GBM) is the most aggressive primary brain tumor. Current treatments, including surgery, radiotherapy, and temozolomide (TMZ), provide only limited benefits, with a median survival of approximately 14–15 months. Consequently, researchers have explored natural compounds, including EVOO and its phenolic constituents, such as oleuropein, HT, and rutin, for their potential anti-glioma effects. Several studies indicate that phenolic compounds derived from olive leaves significantly inhibit GBM cell proliferation through multiple mechanisms [88,89].

#### 5.10.2. Preclinical Evidence

Oleuropein induces apoptosis and growth arrest in GBM cell lines and significantly reduces cell viability. Its activity is comparable to that of whole olive leaf extract, which also suppresses the proliferation of “stem-like” GBM cells characterized by elevated expression of markers such as CD133 and OCT4 [90,91]. In combination therapy, oleuropein administered with TMZ significantly enhanced the therapeutic effect of TMZ, partly through upregulation of Let-7d expression compared with olive leaf extract combined with TMZ [90]. HT also inhibited cell viability, glioma stem cell phenotype, and migration, although its independent anti-GBM activity was less pronounced than that of the total olive leaf extract [91].

Olive leaf extract has been shown to regulate key molecular mediators of EMT. Specifically, olive leaf extract downregulated the mesenchymal marker *N*-cadherin and increased the epithelial marker E-cadherin. These findings align with the well-documented anticancer effects of olive phenolics, which include inhibition of proliferation and modulation of multiple signaling pathways [54,92]. HT significantly reduced GBM cell viability, migration, and the stem-like phenotype. Rutin also altered cancer cell behavior by decreasing intracellular ROS levels and inhibiting colony formation and migration, whereas tyrosol showed minimal biological activity. These findings suggest that HT and rutin contribute substantially to the antiproliferative effects of EVOO, while tyrosol plays a minor role [89].

EVOO phenolics also inhibit invasive behavior in GBM cells. HT and rutin significantly reduced GBM cell motility. At the molecular level, phenol-rich olive leaf extract suppressed key drivers of EMT by inhibiting transcription factors such as Twist, Snail, and Zeb1, as well as reducing *N*-cadherin expression. In addition, combined treatment with olive leaf extract and TMZ increased E-cadherin levels while further reducing mesenchymal markers, indicating inhibition of EMT and decreased tumor invasiveness [93].

#### 5.10.3. In Vivo and Translational Interpretation

EVOO phenolics also enhance the efficacy of conventional chemotherapy. All tested compounds, including oleuropein, HT, tyrosol, and rutin, demonstrated significant additive cytotoxic effects when combined with TMZ in GBM cells (*p* < 0.001) [89]. Co-treatment with olive leaf extract and TMZ significantly modified EMT markers, increasing epithelial E-cadherin and decreasing mesenchymal *N*-cadherin and Twist/Zeb1 expression compared with TMZ treatment alone. These findings suggest that EVOO phenolics not only exert direct cytotoxic effects but also increase tumor cell sensitivity to chemotherapy [93]. However, the ability of olive-derived compounds to enhance TMZ efficacy, improve brain delivery, or prolong survival in patients has not been established.

Researchers have also explored EVOO phenolics in models of brain metastasis. In one study, oleuropein was delivered using pH-sensitive niosomes (Tween 60/cholesterol vesicles) in a rat model of brain metastasis using 4T1 mammary carcinoma cells to facilitate penetration of the blood–brain barrier. In vitro experiments demonstrated that oleuropein-loaded niosomes (Oleu-Nio) significantly reduced 4T1 cell viability (IC_50_ ≈ 92.7 μg/mL), whereas free oleuropein showed minimal cytotoxicity. In vivo, ten intravenous administrations of Oleu-Nio (25 mg/kg) significantly prolonged survival. The median survival exceeded 45 days in treated rats, compared with approximately 34.5 days for free oleuropein and only 16.5 days in untreated controls [94]. These results indicate that appropriate formulation of EVOO phenolics, particularly oleuropein, may enhance therapeutic delivery to the brain, inhibit metastatic tumor growth, and significantly improve survival in preclinical models.

### 5.11. Other Cancers

Evidence for the cancers grouped in this subsection is limited and demonstrates context-dependent effects. Table 4 includes studies of phenolic-rich extracts, isolated phenolics, whole olive-oil dietary interventions, and isolated oleic acid. These exposures are presented separately because they differ substantially in composition and biological activity. Although most phenolic studies report inhibitory effects in experimental cancer models, some fatty-acid and high-fat dietary interventions have promoted tumor growth. Table 4 therefore summarizes both antitumor and pro-tumor findings rather than implying uniformly beneficial activity.

The opposing findings in Table 4 emphasize that EVOO-derived exposures should not be treated as a single biological entity. Phenolic compounds, such as HT, oleuropein, oleocanthal, and oleacein, have generally demonstrated antiproliferative and pro-apoptotic activity in preclinical models. In contrast, isolated oleic acid and high-fat olive-oil-containing diets have produced both inhibitory and stimulatory effects depending on the cancer type, experimental model, dose, and dietary context. Particularly in cervical cancer models, a high-fat olive-oil diet increased HeLa xenograft growth through proliferation-related signaling [98], while isolated oleic acid promoted cervical cancer progression through CD36–Src/ERK signaling [99]. Conversely, oleic acid inhibited endometrial cancer growth through PTEN and AKT/mTOR regulation [100]. These results indicate context-dependent fatty-acid biology and do not contradict the phenolic findings because the investigated exposures, concentrations, and mechanisms differ. Accordingly, conclusions regarding isolated phenolics, isolated oleic acid, and whole EVOO should be reported separately.

## 6. Human Clinical and Translational Evidence

Although considerable mechanistic research has been carried out in cell culture and animal models, there are few direct human studies on the anticarcinogenic activity of extra virgin olive oil or its phytochemical components. Current human evidence is primarily from dietary prevention studies, small exploratory interventions examining hematological markers, and observational studies examining cancer incidence and mortality. Since these various types of evidence are all distinct from one another, it is important to note that changes in circulating biomarkers or signaling molecules related to cancer do not necessarily imply tumor regression, prevention of new cancers, or increased patient survival.

The most relevant randomized evidence was obtained from a prespecified secondary analysis of the PREDIMED trial. The study included 4152 women who were assigned to one of the following groups: a Mediterranean diet with EVOO, a Mediterranean diet with mixed nuts, or a control diet with advice on cutting down on dietary fat. After a median follow-up of 4.8 years, 35 cases of invasive breast cancer were confirmed. Incidence rates were 1.1, 1.8, and 2.9 cases per 1000 person-years, respectively, for the Mediterranean diet plus EVOO, Mediterranean diet plus nuts, and control groups. The multivariable-adjusted hazard ratio (HR) for breast cancer was 0.32 (95% confidence interval [CI]: 0.13–0.79) in the Mediterranean diet plus EVOO group compared with the control group. In a Mediterranean-diet intervention, a secondary analysis reported a lower breast cancer incidence estimate with increasing EVOO intake, with a hazard ratio of 0.72 (95% CI: 0.57–0.90) for each 5% increment in the contribution of EVOO to total energy intake [45]. This result should be interpreted cautiously. Breast cancer incidence was a secondary outcome, the number of cases was limited, and the intervention combined EVOO supplementation with broader Mediterranean diet advice. Therefore, the observed association cannot be attributed specifically to EVOO, and the analysis does not establish a dose-dependent causal reduction in breast cancer risk. The finding requires confirmation in adequately powered trials in which cancer incidence is a prespecified primary outcome and EVOO is evaluated independently against an appropriate comparator oil.

The effectiveness of high-oleocanthal and high-oleacein EVOO was evaluated in a pilot dietary intervention of patients with untreated early-stage CLL. The first blinded randomized phase involved patients with Rai stage 0–II CLL taking EVOO containing either high or low levels of oleocanthal and oleacein, at 40 mL/day for three months. After a 9–12-month washout phase, the second trial involved 22 patients who took 40 mL/day of high-oleocanthal/high-oleacein EVOO for six months. Consumption of EVOO with a high phenolic content was linked to decreases in white blood cells and lymphocytes, increased expression of apoptosis markers, cleaved cytokeratin-18 and Apo1-Fas, and increases in the expression of the cell-cycle inhibitor p21, as well as decreases in survivin and cyclin D expression. The results obtained represent preliminary evidence of the ability of a chemically characterized EVOO preparation with high phenolic content to modulate disease-related hematological and molecular biomarkers in humans. However, the study was small, did not have a concurrent control group, and the measured outcomes were surrogate rather than objective measures of tumor response, time to treatment, time to progression, or overall survival [87].

Prospective observational evidence links olive oil intake with cancer mortality. In the Moli-sani cohort, 22,892 Italian adults were followed for a median of 13.1 years. Additionally, olive oil consumption of more than 3 tablespoons/day was associated with reduced cancer mortality compared with 1.5 tablespoons/day or less (multivariable-adjusted hazard ratio, 0.77; 95% CI: 0.59–0.99). The overall contribution of the inflammatory, metabolic, cardiovascular, and renal markers was responsible for around 13.7% of the overall relationship between olive oil consumption and cancer mortality. However, this study measured total olive oil and not chemically defined EVOO, and due to its observational study design, residual confounding, reverse causality, and the effect of the overall dietary and lifestyle pattern could not be ruled out [104].

Overall, the human evidence available thus far suggests a potential role of EVOO in cancer prevention [87] and supports the biological activity of high-phenolic EVOO for specific patient populations [45,104]. At present, there is no evidence that EVOO or individual olive oil phenolics are useful as cancer treatments. There is limited and inconclusive clinical evidence for objective tumor response, cancer recurrence, disease-free survival, progression-free survival, overall survival, or clinically meaningful interactions with chemotherapy, radiotherapy, targeted therapy, or immunotherapy. Chemically characterized EVOO preparations, standardized doses of phenolics, appropriate control oils, pharmacokinetic parameters, adequate sample sizes, sufficient follow-up periods and clinically relevant oncological parameters should be used in future trials.

## 7. Nanotechnology and Delivery Strategies

Nanotechnology has emerged as a central strategy for delivering olive oil phenolics to the brain. Lipid-based nanostructured carriers that retain a pure olive oil core demonstrate significantly higher in vitro blood–brain barrier (BBB) translocation compared with more rigid formulations containing stearic acid, highlighting the importance of a soft, oleic acid-rich matrix for efficient brain uptake [141]. Surface modification further enhances BBB penetration: PEGylation prolongs systemic circulation, while targeting ligands such as transferrin, angiopep-2, or folic acid facilitate receptor-mediated transcytosis, thereby improving nanoparticle accumulation in the brain [142].

These design strategies have been applied to the delivery of phytochemicals for GBM treatment. A 2024 review emphasized that encapsulating anticancer phytochemicals, such as oleuropein and HT, in nanocarriers such as solid lipid nanoparticles, nanostructured lipid carriers, polymeric nanoparticles, and mesoporous silica can overcome poor BBB permeability and tumor microenvironment barriers. Such systems enhance apoptosis induction, reduce stem cell markers, and inhibit EMT in GBM models [143,144]. Polymeric nanoparticles such as PLGA and PLA provide tunable degradation and surface functionalization, enabling sustained drug release and multimodal therapeutic strategies, while demonstrating effective BBB penetration and prolonged drug exposure in glioma models [145].

Recent studies involving lipid nanoparticles have confirmed that nanoencapsulation of phenolic compounds improves their physiological stability, enhances BBB permeability, and produces stronger neuroprotective effects compared with free compounds [146]. Collectively, these advances indicate that engineered nanocarriers, whether lipid-based, polymeric, or inorganic, can significantly enhance the therapeutic potential of oleuropein and HT against primary and metastatic brain tumors. Although nanocarriers have improved stability, cellular uptake, blood–brain barrier penetration, and antitumor activity in experimental systems, these findings remain preclinical. Improved delivery and cytotoxicity do not establish therapeutic benefit, safety, or superiority over free compounds in humans.

## 8. Limitations and Future Perspectives

Although these findings are promising, the clinical relevance of EVOO phenolics is still uncertain. The available evidence is mostly based on isolated HT, oleuropein, oleocanthal, and oleacein at µM concentrations in cell cultures and animal models. These exposures can be higher than what can be attained with typical EVOO intake. Furthermore, EVOO composition depends on cultivar, maturity, processing, storage and geographical origin. Chemically characterized preparations, total phenol content, individual phenol content, fatty-acid composition, oxidation status, administered dose, and pharmacokinetic exposure should be explored in future studies.

Liposomes, niosomes, polymeric nanoparticles, lipid nanoparticles, and mesoporous carriers are examples of nanoformulations that can enhance the stability, solubility, controlled release, tissue distribution, and cellular uptake of phenolics. For tumors with physiological barriers, such as glioblastoma, targeted and stimulus-responsive systems could be especially beneficial. However, future research should be conducted to assess particle characteristics, particle release rates, biodistribution, tumor accumulation, carrier toxicity, long-term stability, manufacturing reproducibility, and scalability. Nano-encapsulated phenolics are not to be considered equivalent products to dietary EVOO, but rather as pharmacological products. Moreover, experimental studies have demonstrated that EVOO phenolics have the potential to augment the efficacy of doxorubicin, 5-fluorouracil, gemcitabine, proteasome inhibitors, and anti-CD47 immunotherapy. Future research is needed to establish whether the interactions are synergistic or additive, to identify clinically relevant doses, to assess the pharmacokinetic interactions, mechanisms of resistance, and toxicity in normal tissues, and to evaluate treatment sequence. Special consideration must be given because some olive phenolics can be antioxidants or pro-oxidants in certain circumstances at specific doses, which can either promote or hinder standard treatments.

EVOO response can vary by age, sex, genetics, metabolic status, prior diet, inflammatory status, drug use, cancer type, and phenol-metabolizing ability. Subgroups of participants who are likely to benefit from phenolic-rich EVOO should be determined in precision nutrition studies. The amount of consumed food should be evaluated multiple times and, if possible, complemented with plasma and urinary markers of olive phenolic exposure. Furthermore, the gut microbiome could convert EVOO phenolics into metabolites that have different absorption profiles and bioactivities. On the other hand, the constituents of EVOO can affect the composition of the microbiota, intestinal barrier function, inflammatory responses, immune regulation, and bile-acid metabolism. Future studies should incorporate measurements of plasma, urine, and fecal phenolic metabolites with dietary assessment, metabolomics, and metagenomic analyses. It is important to control medication use, baseline microbiome composition, and geography, as these can significantly impact microbial responses. The safety of these substances is crucial before their implementation in functional foods and nutraceutical applications [147].

Current studies focus only on a few signaling proteins. Genomics, epigenomics, transcriptomics, proteomics, metabolomics, lipidomics, and microbiome profiling may have a more comprehensive coverage of the effects related to EVOO. Single-cell and spatial methods might be useful to differentiate the immune-cell, fibroblast, endothelial cell, and tumor cell responses. The results of omics studies should not be interpreted as proof of clinical efficacy but should be correlated with pharmacokinetic data and validated in targeted molecular experiments. Chemically characterized EVOO and specific formulations of phenolics, suitable comparator oils, predetermined outcomes, and objective adherence measures should be used in future trials. Safety, biomarkers of plasma and tissue exposure, and target engagement should be evaluated in early-phase studies, including plasma and tissue phenolic metabolites, markers of cell proliferation (Ki-67, cleaved caspase-3), markers of cell activation (phosphorylated AKT or STAT3), inflammatory cytokines, circulating tumor DNA, and circulating tumor cells. Future trials need to measure clinically relevant endpoints, such as lesion progression, pathological response, treatment toxicity, recurrence, progression-free survival, and overall survival. In general, future studies should differentiate between dietary EVOO and pharmacological formulations of phenolics, and focus on realistic doses, standardized preparations, pharmacokinetic studies, cancer-specific models, and combination studies, including rigorous human studies, microbiome-informed precision nutrition, integrated multi-omics, and biomarker-guided clinical trials.

Beyond biological efficacy, commercialization of EVOO-derived phenolics should consider sustainability and intellectual property. Plant-patent systems can encourage innovation in olive breeding and bioactive product development but may also limit access to genetic resources. Iftikhar et al. highlighted the need to balance innovation with biodiversity conservation, farmers’ rights, food security, and equitable benefit-sharing [148]. These principles should guide the sustainable development and commercialization of EVOO-derived products.

## 9. Conclusions

Existing evidence supports two related but distinct conclusions. First, observational and limited dietary evidence suggests that olive oil or EVOO intake may be associated with a reduced risk of certain cancers in some populations, although confounding by the overall Mediterranean dietary pattern and other lifestyle factors remains possible. Second, most detailed anticancer mechanisms, including regulation of PI3K/AKT/mTOR, MAPK/ERK, NF-κB, JAK/STAT, Wnt/β-catenin, p53, apoptosis, angiogenesis, and EMT, have been demonstrated using isolated HT, oleuropein, oleocanthal, oleacein, and phenolic extracts in preclinical models. Because the administered doses, bioavailability, metabolism, and matrix interactions differ substantially from dietary EVOO exposure, compound-specific findings should not be directly extrapolated to whole-EVOO consumption or clinical efficacy. Moreover, throughout this review, terms such as “induces,” “inhibits,” “enhances,” and “protects” refer only to the experimental model in which the effect was observed. Current evidence does not establish that EVOO phenolics enhance anticancer therapy, selectively protect healthy tissues, prevent recurrence, or improve survival in humans. Their clinical value remains to be determined through pharmacokinetic investigations and controlled trials with validated oncological endpoints.

## Figures and Tables

**Table 1:** Comparative summary of evidence for EVOO, olive-derived preparations, and isolated phenolics across cancer types.

Cancer Type	Model	EVOO Constituent or Preparation	Dose	Molecular Targets	Outcomes	Evidence Level	References
Breast	MDA-MB-231, MCF-7, TNBC and HER2-positive breast-cancer cells	Oleocanthal and oleuropein	NR	HGF/Met, PI3K/AKT/mTOR, MAPK/ERK, NF-κB, cyclin D1, p21, caspase-8, caspase-3 and PARP	Cell-cycle arrest; reduced proliferation, migration and invasion; increased apoptosis	In vitro	[37,38,39]
Breast	MMTV-PyVT transgenic mouse model	Oleocanthal	7.5 mg/kg/day orally for 32 days	Tumor-initiation and growth-related pathways	Approximately 70% suppression of tumor initiation and progression	Animal	[40]
Breast	MDA-MB-231 xenograft model	Oleocanthal	5 mg/kg	p-cMet, Ki-67, CD31, vimentin, β-catenin, HER2 and E-cadherin	Reduced tumor growth, proliferation, invasion, EMT and angiogenesis	Animal	[41,42]
Breast	BT-474 HER2-positive tumor model	Oleocanthal	Daily administration; dose NR	HER2- and estrogen-dependent growth pathways	Reduced estrogen-driven tumor growth and locoregional recurrence after surgery	Animal	[43]
Breast	MDA-MB-231 xenograft treated with doxorubicin	Oleuropein plus doxorubicin	NR	NF-κB, cyclin D1, Bcl-2 and surviving	Increased apoptosis and enhanced activity of doxorubicin	Animal	[44]
Breast	PREDIMED secondary analysis; 4152 women without previous breast cancer	Mediterranean diet supplemented with EVOO	Exact EVOO dose NR in the manuscript; intake also analyzed per 5% of total energy	Molecular targets not measured	Lower breast-cancer incidence estimate compared with the control diet; cancer was a secondary outcome with few events	Human-interventional secondary analysis	[45]
Prostate	LNCaP and DU145	Oleuropein	100–500 µM	AKT phosphorylation	Reduced proliferation through AKT dephosphorylation	In vitro	[46]
Prostate	22Rv1, LNCaP, PC-3, and C4-2	HT	IC_50_ values approximately 9–41 µM	Caspase-3, caspase-9, PARP, cyclins D1/E, CDK2/4, Bax, Bcl-2 and Bcl-xL	G_1_-phase arrest, intrinsic apoptosis and selective toxicity toward malignant cells	In vitro	[9,47,48]
Prostate	PC-3 and 22Rv1	HT and analogs	NR	Migration- and stemness-related signaling	Reduced PC-3 migration and prostatosphere formation in 22Rv1 cells	In vitro	[49]
Colorectal	HT-29 and other colorectal-cancer cells	HT	NR	CDK1, cyclin B1, caspase-3, Bax, EGFR and PI3K/AKT	G_2_/M-phase arrest, apoptosis and reduced proliferation	In vitro	[50,51,52,53]
Colorectal	HT-29	Oleuropein	NR	HIF 1α, p53, NF-κB, AKT, COX-2 and Wnt/β-catenin	Reduced proliferation, DNA fragmentation and mitochondrial apoptosis	In vitro	[54,55,56,57]
Colorectal	COLO-320DM	Oleocanthal	IC_50_ approximately 9.8 µM	Proliferation- and survival-related signaling	Strong inhibition of colorectal-cancer cell proliferation	In vitro	[58]
Colorectal	Mouse colorectal-tumor xenograft and recurrence models	Food-grade oleocanthal or high-phenolic EVOO	NR	Tumor-growth and recurrence-related pathways	More than 70% reduction in tumor burden and more than 85% inhibition of relapse	Animal	[59]
Pancreatic	MIA PaCa-2; 72 h	Oleuropein	IC_50_ approximately 150 µM	c-Jun, c-Fos and caspase-3	Reduced viability, G_2_/M-phase arrest and apoptosis	In vitro	[46]
Pancreatic	MIA PaCa-2; 72 h	HT	IC_50_ approximately 75 µM	c-Jun, c-Fos and caspase-3	Reduced viability, G_2_/M-phase arrest and apoptosis	In vitro	[46]
Pancreatic	PANC-1; 24–72 h	HT	10–320 µM; apoptosis observed at ≥19 µM	Caspase-9, Bax, MMP-2 and MMP-9	Dose- and time-dependent loss of viability, increased apoptosis and reduced invasion-associated markers	In vitro	[60]
Pancreatic	Panc02; 48 h	HT	50–200 µM	Phospho-STAT3 and cyclin D1	Reduced proliferation and increased apoptosis	In vitro	[60]
Pancreatic	Three-dimensional PDAC stromal co-culture	Oleocanthal	20 µM	Lysosomal membrane permeabilization, cathepsins, αSMA-positive CAFs, hyaluronan, IL-6/STAT3 and COX-2/mPGES-1	Direct PDAC-cell death; approximately 50% reduction in activated fibroblasts and 70% degradation of the hyaluronan matrix	In vitro-3D co-culture	[42]
Pancreatic	Mouse pancreatic ductal adenocarcinoma model treated with anti-CD47	HT plus anti-CD47	NR	Myeloid-derived suppressor cells, M1 macrophages and immune-microenvironment signaling	Enhanced antitumor activity of anti-CD47 immunotherapy	Animal	[60]
Pancreatic	Transgenic pancreatic neuroendocrine-tumor model	Oleocanthal	NR	Tumor-growth and survival pathways	Suppressed tumorigenesis and prolonged survival	Animal	[61,62]
Bone	MG-63 human osteosarcoma cells	Oleuropein alone or combined with adriamycin	IC_50_ = ~22 µg/mL	G_0_/G_1_ cell-cycle regulation and cell-death-related responses	Reduced proliferation and greater cytotoxic activity when combined with Adriamycin	In vitro	[63,64]
Bone	Osteosarcoma models treated with adriamycin	Oleuropein plus adriamycin	NR	Apoptotic and autophagic pathways	Synergistic increase in tumor-cell cytotoxicity	Preclinical/animal	[55,63]
Oral	KB oral-cancer and HNO-97 tongue-cancer cells	HT and oleuropein	NR	Cyclins, CDKs, Bax, Bcl-2, caspases, NF-κB, COX-2, PI3K/AKT and MAPK	G_0_/G_1_-phase arrest, apoptosis and reduced migration and wound closure	In vitro	[65,66,67,68]
Oral	Experimental tongue-lesion model	Olive-derived phenolic preparation	NR	Inflammatory and apoptosis-related pathways	Reduced gross and histological tongue lesions	Animal	[69]
Oral/head and neck	Meta-analysis and observational dietary studies	Mediterranean dietary pattern containing olive oil	Dietary exposure; dose varied	Molecular targets not directly measured	Higher Mediterranean-diet adherence was associated with lower odds of oral, head and neck cancers	Human-observational	[70,71,72]
Liver	Italian case–control, pooled and meta-analytic studies of HCC	Olive oil, EVOO or Mediterranean dietary pattern	Approximately 20 g/day in some case–control analyses; other studies used intake categories	Molecular targets not directly measured; proposed oxidative-stress, inflammation and lipid-metabolism pathways	Higher intake was associated with lower relative HCC-risk estimates in some studies; findings were inconsistent across populations	Human-observational	[73,74]
Gastric	Gastric adenocarcinoma cells	Oleuropein, HT, oleocanthal or phenolic-enriched extracts	Oleuropein 50–500 µM; other doses NR	ROS, p53, Bax, Bcl-2, caspases, PI3K/AKT/mTOR, NF-κB, Wnt/β catenin, EGFR and c-Met	Reduced viability, cell-cycle arrest and mitochondrial apoptosis	In vitro	[54,75,76,77,78]
Gastric	AGS gastric-cancer cells	Methanolic Greek table olive extract	NR	ICAM-1, IL-8, inflammatory and apoptotic signaling	Reduced proliferation and inflammation and increased apoptosis	In vitro	[79]
Gastric	*Helicobacter pylori* strains, including resistant strains	EVOO or olive oil phenolic preparations	Minimum inhibitory concentration approximately 230 µg/mL	Bacterial adhesion and infection-induced epithelial DNA damage	Anti-H. pylori activity and reduced adhesion and epithelial injury	In vitro	[80,81]
Gastric	*Helicobacter pylori*-infected mouse model	Dietary EVOO supplementation	NR	Gastric inflammation and mucosal-injury pathways	Reduced ulcer indices and gastric mucosal lesions and improved histological appearance	Animal	[80,81]
Gastric/gastrointestinal	Italian observational cohort	EVOO intake	Intake categories; exact dose NR	Molecular targets not measured	Higher EVOO intake was associated with lower gastrointestinal cancer-mortality estimates	Human-observational	[74]
Blood leukemia	HL-60 acute promyelocytic leukemia cells	EVOO phenolic extract or HT	NR	CDK6, p21, p27, cytochrome c and caspase-3	G_0_/G_1_-phase arrest, reduced proliferation and apoptosis	In vitro	[82,83]
Blood CML	K562 chronic myeloid leukemia cells	Oleuropein	IC_50_ approximately 244 µM at 72 h	Redox homeostasis, p21, p27 and cyclins	Time- and dose-dependent reduction in viability and cell-cycle arrest	In vitro	[84]
Blood leukemia/lymphoma	Jurkat, CEM, Raji and K562 cells	Oleocanthal	Approximately 5–30 µM	Mitochondrial membrane potential, ROS, caspase-3/8/9, AKT and ERK1/2	Reduced proliferation and selective apoptosis with lower toxicity toward non-malignant cells	In vitro	[85]
Blood multiple myeloma	Multiple myeloma cells treated with a proteasome inhibitor	Oleacein	NR	Caspase-8, Sp1 and HDAC1/2/3/4/6	Histone hyperacetylation and increased sensitivity to proteasome-inhibitor therapy	In vitro	[86]
Blood CLL	Untreated Rai stage 0–II CLL patients	Chemically characterized high-oleocanthal/high-oleacein EVOO	40 mL/day for 3 months; subsequent study, 40 mL/day for 6 months in 22 patients	Cleaved cytokeratin-18, Apo1-Fas, p21, survivin and cyclin D	Reduced white-blood-cell and lymphocyte counts and modulation of apoptotic and cell-cycle biomarkers; no established effect on progression or survival	Human-pilot intervention	[87]
Brain glioblastoma	GBM and glioma stem-like cells	Olive leaf extract, oleuropein, HT, rutin and tyrosol	NR	CD133, OCT4, Let-7d, E-cadherin, *N*-cadherin, Twist, Snail and Zeb1	Reduced viability, stemness, colony formation, migration and EMT; enhanced temozolomide activity	In vitro	[88,89,90,91,92,93]
Brain metastasis	4T1 mammary carcinoma cells	Oleuropein-loaded pH-sensitive niosomes	IC_50_ approximately 92.7 µg/mL	Formulation-dependent cellular delivery and viability pathways	Greater cytotoxicity than free oleuropein	In vitro	[94]
Brain metastasis	Rat 4T1 brain metastasis model	Oleuropein-loaded pH-sensitive niosomes	25 mg/kg intravenously; 10 administrations	Brain delivery and tumor-growth pathways	Median survival greater than 45 days versus approximately 34.5 days with free oleuropein and 16.5 days in untreated controls	Animal	[94]
Cervical	HeLa	Olive extract	50 µg/mL	p21/CDKN1A and caspase-3	Reduced proliferation and colony formation and increased apoptosis	In vitro	[95]
Cervical	HeLa; 48–72 h	Oleuropein	Approximately 10–100 µM	Bcl-2, Mcl-1, Bid, Fas, TNFRSF10B, p53 and pro-apoptotic microRNAs	Reduced viability through apoptosis	In vitro	[96]
Cervical	HeLa, SiHa, and HCS-2	Different EVOO preparations	NR	HPV E6/E7, p16, p63, involucrin and miR-331	Reduced viability and HPV-oncogene expression and increased differentiation markers	In vitro	[97]
Cervical	HeLa xenograft in nude mice	High-fat diet containing olive oil	45% of energy from fat	EGR1 and oleic-acid-responsive proliferative signaling	Increased tumor growth and tumor weight relative to the control diet	Animal	[98]
Cervical	HeLa and xenograft model	Oleic acid	NR	CD36, Src and ERK	Increased proliferation, migration, invasion, tumor growth and metastasis	In vitro and animal	[99]
Endometrial	Endometrial-cancer cells and an in vivo model	Oleic acid	NR	PTEN and AKT/mTOR	G_1_ arrest, apoptosis, reduced proliferation and invasion and approximately 52% reduction in tumor size	In vitro and animal	[100]
Ovarian	HEY	Oleuropein	NR	Fe^2+^, ROS and cell-cycle pathways	Dose-dependent reduction in viability and apoptosis at higher concentrations	In vitro	[68]
Melanoma	Human melanoma cell lines	HT	NR	ROS, γH2AX, p53, AKT, caspase-3 and PARP	Dose-dependent apoptosis and inhibition of colony formation	In vitro	[47]
Melanoma	Human melanoma cell lines	Oleocanthal	IC_50_ in the low-µM range	ERK1/2, AKT and Bcl-2	Selective cytotoxicity and apoptosis	In vitro	[101]
Skin squamous-cell carcinoma	Human non-melanoma SCC cell models	Oleocanthal and oleacein	NR	Phospho-ERK, phospho-AKT and B-Raf	Reduced viability, motility and colony/spheroid formation and increased apoptosis	In vitro	[102]
Thyroid	TPC-1 and FB-2 thyroid-cell models	HT	NR	p21, cyclin D1, ROS and mitochondrial-apoptosis pathways	Reduced viability, cell-cycle arrest and apoptosis	In vitro	[103]
Multiple cancer outcomes	Moli-sani prospective cohort; 22,892 adults	Total olive oil	>3 tablespoons/day versus ≤1.5 tablespoons/day	Molecular targets not directly measured; inflammatory, metabolic, cardiovascular and renal biomarkers were explored as possible mediators	Higher intake was associated with lower cancer-mortality estimates; residual confounding and reverse causality cannot be excluded	Human-observational	[104]

AKT, protein kinase B; CAF, cancer-associated fibroblast; CLL, chronic lymphocytic leukemia; CML, chronic myeloid leukemia; COX-2, cyclooxygenase-2; EMT, epithelial–mesenchymal transition; EVOO, extra virgin olive oil; GBM, glioblastoma; HDAC, histone deacetylase; HCC, hepatocellular carcinoma; HT, hydroxytyrosol; NR, not reported in the reviewed manuscript; OCT4, octamer-binding transcription factor 4; PDAC, pancreatic ductal adenocarcinoma; ROS, reactive oxygen species; SCC, squamous-cell carcinoma; STAT3, signal transducer and activator of transcription 3; TNBC, triple-negative breast cancer.

**Table 2:** In vitro and in vivo efficacy of EVOO phenolics against pancreatic carcinoma.

Phenolic Compound	Model and Dose	Mechanisms	References
Oleuropein	MIA PaCa-2 cells (72 h)	Reduced viability (IC_50_ = 150 μM), G_2_/M phase arrest, increased c-Jun/Fos, caspase-3 mediated apoptosis	[46]
Hydroxytyrosol	MIA PaCa-2 cells (72 h)	Reduced viability (IC_50_ ≈ 75 μM), G_2_/M phase arrest, increased c-Jun/Fos, caspase-3-mediated apoptosis	
	PANC-1 (24–72 h, 10–320 µM)	Dose- and time-dependent reduction in viability, apoptosis at concentrations ≥ 19 μM, increased caspase-9/Bax expression and decreased MMP-2/9 RNA levels	
	Panc02 (48 h, 50–200 μM)	Decreased proliferation, increased apoptosis, reduced phospho-STAT3 and Cyclin D1	
Oleocanthal	Three-dimensional pancreatic ductal adenocarcinoma stromal co-culture (20 µM)	Approximately 50% reduction in α-SMA^+^ CAFs, approximately 70% degradation of the hyaluronan matrix, and increased CD8^+^ T-cell density	[42]
	PNET: dosage in transgenic murine models	Significant suppression of pancreatic neuroendocrine tumor proliferation, extended survival	

Abbreviations: CAF, cancer-associated fibroblast.

**Table 3:** In vitro studies related to the phytochemicals in blood cancer management.

Extract	Cell Lines	Concentration	Findings	Mechanisms	Refs.
Oleocanthal	Jurkat (T-ALL), CEM (T-ALL), Raji (Burkitt), and K-562 (CML)	5–30 μM (approx.)	Decreased proliferation across all tested cell lines (except lung cells) and significant apoptosis.	Caspase-dependent and -independent apoptosis, increased reactive oxygen species, mitochondrial depolarization.	[85]
Oleacein	JJN3, U266, and OPM2 multiple myeloma cells	~10–40 μM	Decreased viability, increased apoptosis, and increased sensitivity to carfilzomib	Caspase-8-associated Sp1 downregulation, reduced HDAC1/2/3/4/6 expression, and increased histone acetylation	[86]
Oleuropein	K-562 (CML)	IC_50_ ≈ 244 μM at 72 h	Reduced viability, elevated apoptosis, and possibly synergy with carfilzomib.	Pro-oxidant effect: ↑ ROS, ↓ antioxidant defenses, possible DNA damage (8-OHdG ↑).	[84]

↑, increase; ↓, decrease.

**Table 4:** Context-dependent effects of olive-derived phenolics, oleic acid, and olive-oil-containing dietary interventions in exploratory cancer models.

Model System	Exposure Category	Compounds and Dosage	Main Findings	Mechanistic Targets	References
HeLa	Phenolic extract	Olive extract (50 μg/mL)	Decreased proliferation and colony formation, increased caspase-3 activity	Increase of p21 (CDKN1A) (marker of apoptosis)	[95]
HeLa	Isolated phenolic	Oleuropein (~10–100 μM for 48–72 h)	Decreased viability through apoptosis, increased pro-apoptotic microRNAs and genes	Decreased levels of Bcl-2 and Mcl-1, increased levels of Bid, Fas, TNFRSF10B, and p53	[96]
HeLa	Phenolic extract	OLE (phenol-rich)	Decreased viability, cell cycle arrest, activation of apoptosis	Decreased Cyclin D1, increased p21, decreased NF-κB/EMT	[122]
HeLa/SiHa/HCS-2 (in vitro)	Whole-oil dietary intervention	Extra-virgin olive oils	Decreased viability and ROS, decreased HPV E6/E7, p16, p63, increased differentiation marker (IVL)	Downregulation of E6/E7 oncogenes, increased expression of tumor-suppressive miR-331	[97]
HeLa	Phenolic extract	Olive leaf polyphenols (nanocarrier) and NDV (LaSota), OLE 100 μM (oleuropein equiv) and NDV MOI 0.4	Synergistic cytotoxicity (increased cell death), cell cycle arrest	Induced apoptosis, increased OLE absorption by nanocarriers and viral oncolysis	[139]
Women with external anogenital warts	Combination	OLE and curcumin (topical 10%) administered three times daily (for a maximum of twelve weeks)	Reduction in the number of warts (from day 5, *p* = 0.027), reduction in healing time (14.7 vs. 34.3 days, *p* = 0.001)	Expedited lesion regression (HPV eradication)	[140]
HeLa xenografts in nude mice	Whole-oil dietary intervention	High-fat diet containing olive oil; 45% of energy from fat	Increased tumor growth and weight relative to the control diet	Increased EGR1 and other proliferation-related signals	[98]
Endometrial-cancer cells and an in vivo model	Isolated fatty acid	OA	G_1_-phase arrest, increased apoptosis, reduced proliferation and invasion, and approximately 52% reduction in tumor size	Increased PTEN and reduced AKT/mTOR signaling	[100]
Ovarian HEY cells	Isolated phenolic	Oleuropein (olive leaf polyphenol)	Pro-oxidant at elevated dosage: increased Fe^2+^, elevated ROS, cell-cycle inhibition	Dose-dependent decrease in viability, high doses trigger apoptosis.	[68]
HeLa cervical cancer cells and xenograft model	Isolated fatty acid	OA	Increased proliferation, migration, invasion, tumor growth, and metastasis	CD36-mediated fatty-acid uptake and activation of Src/ERK signaling	[99]
In vitro human melanoma cell lines	Isolated phenolic	HT	Increased ROS generation, elevated DNA damage (γH2AX) and p53, decreased Akt signaling induces caspase-3/PARP-mediated apoptosis	Dosage-dependent apoptosis in melanoma cells, resulting in the cessation of colony formation	[101]
In vitro human melanoma cell lines	Isolated phenolic	Oleocanthal	Decreased phosphorylation of ERK1/2 and Akt, reduced levels of Bcl-2, which inhibits cellular apoptosis, likely impedes the MAPK and PI3K signaling pathways	Selective cytotoxicity against melanoma (IC_50_ in low μM) accompanied by apoptosis induction	[101]
Skin squamous cell carcinoma in humans (in vitro; non-melanoma varieties)	Isolated phenolic	Oleocanthal, oleacein	Reduce Phospho-ERK and phospho-Akt, diminish B-Raf expression (inhibits MAPK/Akt signaling)	Impaired SCC cell viability, motility, and colony/spheroid formation, increased apoptosis.	[102]
The TPC-1 and FB-2 human peritoneal and fibroblast cell lines (in vitro)	Isolated phenolic	HT	Cell cycle arrest, intrinsic mitochondrial apoptosis, and p21/Cyclin D1, stress caused by pro-oxidants (H_2_O_2_/ROS)	Decreased cell viability, increased apoptosis	[103]

Abbreviations: EMT, epithelial–mesenchymal transition; HT, hydroxytyrosol; OA, oleic acid; OLE, olive leaf extract; ROS, reactive oxygen species.

## Data Availability

No new experimental, clinical, animal, or participant-level data were generated in this study. All bibliometric information was derived from published literature as described in the Methods. Bibliographic records obtained from Web of Science and Scopus remain subject to the respective database access conditions.

## Abbreviations

The following abbreviations are used in this manuscript:

BBB	Blood–brain barrier
CDKs	Cyclin-dependent kinases
CLL	Chronic lymphocytic leukemia
CML	Chronic myeloid leukemia
CRC	Colorectal cancer
EMT	epithelial–mesenchymal transition
EVOO	Extra virgin olive oil
GBM	Glioblastoma
HCC	Hepatocellular carcinoma
HT	Hydroxytyrosol
Oleu-Nio	Oleuropein-loaded niosomes
PDAC	pancreatic ductal adenocarcinoma
TMZ	Temozolomide
TNBC	Triple-negative breast cancer
